# Protein functional features are reflected in the patterns of mRNA translation speed

**DOI:** 10.1186/s12864-015-1734-7

**Published:** 2015-07-09

**Authors:** Daniel López, Florencio Pazos

**Affiliations:** Computational Systems Biology Group, National Centre for Biotechnology (CNB-CSIC), c/ Darwin, 3, Madrid, 28049 Spain; Current address: BacMine, S.L. c/ Santiago Grisolía, lab 151, Parque Científico de Madrid, Tres Cantos, Madrid, 28760 Spain

## Abstract

**Background:**

The degeneracy of the genetic code makes it possible for the same amino acid string to be coded by different messenger RNA (mRNA) sequences. These “synonymous mRNAs” may differ largely in a number of aspects related to their overall translational efficiency, such as secondary structure content and availability of the encoded transfer RNAs (tRNAs). Consequently, they may render different yields of the translated polypeptides. These mRNA features related to translation efficiency are also playing a role locally, resulting in a non-uniform translation speed along the mRNA, which has been previously related to some protein structural features and also used to explain some dramatic effects of “silent” single-nucleotide-polymorphisms (SNPs). In this work we perform the first large scale analysis of the relationship between three experimental proxies of mRNA local translation efficiency and the local features of the corresponding encoded proteins.

**Results:**

We found that a number of protein functional and structural features are reflected in the patterns of ribosome occupancy, secondary structure and tRNA availability along the mRNA. One or more of these proxies of translation speed have distinctive patterns around the mRNA regions coding for certain protein local features. In some cases the three patterns follow a similar trend. We also show specific examples where these patterns of translation speed point to the protein’s important structural and functional features.

**Conclusions:**

This support the idea that the genome not only codes the protein functional features as sequences of amino acids, but also as subtle patterns of mRNA properties which, probably through local effects on the translation speed, have some consequence on the final polypeptide. These results open the possibility of predicting a protein’s functional regions based on a single genomic sequence, and have implications for heterologous protein expression and fine-tuning protein function.

**Electronic supplementary material:**

The online version of this article (doi:10.1186/s12864-015-1734-7) contains supplementary material, which is available to authorized users.

## Background

The genetic code is said to be “degenerated”, meaning that most of the 20 natural amino acids can be coded by more than one triplet of nucleotides (synonymous codons). The usage of synonymous codons affects the translation process in many ways, in some cases with pathological consequences [1, 2]. For example, the usage of alternative synonymous codons can alter the recognition sites for regulatory micro RNAs and consequently have an effect on the mRNA concentration (e.g. [3]). Similarly, “synonymous mRNAs” (those whose differences are only due to synonymous codons and hence coding for the same polypeptide) can eventually have different secondary and tertiary structures due to the different base pairings formed by their nucleotides. In mRNA a small change in sequence can lead to large structural changes [4]. This can also have effects on their global translation efficiencies since the mRNA has to be unfolded for being scanned by the ribosome [5]. Finally, another consequence of using different synonymous codons for a given amino acid is related to the differential availability (abundance) of the alternative transfer RNAs charged with that particular amino acid (aminoacyl-tRNAs). Different studies showed that, for many amino acids, their synonymous tRNAs differ greatly, both in the number of genes coding for them, and in their cytoplasmic concentration (these two indicators of tRNA abundance are indeed correlated [6]). Consequently, mRNAs enriched in codons associated to abundant tRNAs (termed “optimal codons”) will be translated faster than those using codons associated to tRNAs which are at low concentrations (“rare codons”). Indeed, there is a certain correlation between the relative usage of synonymous codons by an organism and the abundances of the corresponding tRNAs [7–10]. For this reason, proteins which have to be highly expressed are globally enriched in codons associated to abundant tRNAs so that they can be translated faster [11, 12]. This interplay between tRNA abundance and codon usage has been shown to play a central role in the regulation of the overall protein levels in a number of biological systems [13, 14].

So, the differential usage of synonymous codons can affect the overall translation efficiency of a protein through a number of mechanisms and, consequently, affect its final concentration. But at first sight it would not have an obvious effect on the characteristics (structure and function) of its individual molecules, only on their abundance. Nevertheless, the same effect differential codon usage has on the overall translation speed of a protein is also playing a role locally: in principle it would be possible to modulate the translation speed of different regions of a protein chain choosing among the codons available for the amino acids required in these regions (see [15] and references herein). In the light of the currently accepted “co-translational” mechanism for protein folding, according to which the nascent polypeptide folds sequentially as it gets out the ribosome [16], that possibility of slowing down the translation of some parts of the protein while speeding up others could eventually have some effect on its structure and/or function. For example Kimchi-Sarfaty and colleagues found that a single-nucleotide polymorphism (SNP) which does not alter the coding sequence has a drastic effect on the function of the protein without altering the mRNA or protein levels [17]. A possible explanation involves that this “silent” SNP has a local effect on the elongation speed and, consequently, affects the co-translational folding and the proper binding of the protein to its molecular partners. Similarly, Agashe et al. [18] generated different synonymous mRNAs for a bacterial enzyme and showed that not only the amount of produced proteins changed largely, but their relative enzymatic activities as well. This is another indication that the differential usage of synonymous codons can influence the structure/function of the translated proteins, possibly by locally modulating the translation efficiency/speed, and not only on their overall concentration.

A technique called “ribosomal profiling” allows inferring relative translational speeds at the nucleotide resolution, indirectly as the inverse of the ribosomal occupancy [19]. Using this method, Dana and Tuller reported that the translational speed along mRNAs was far from constant and indeed influenced by many local factors such as tRNA availability, predicted mRNA local structure or amino acid charge [20]. For example, the translational speed of the first 20–30 codons of yeast and mammalian mRNAs was lower than the rest of the messenger (translational “ramp”), what could have implications for ensuring a proper attachment of the ribosomes to the mRNA and the starting of the folding process of the nascent polypeptide [6, 20]. This technique also showed that translational pauses are associated to “turns” in the secondary structure of the translated protein [21]. Using this approach it has also been found that translational slowdowns are globally associated to certain types of amino-acids, such as those with positive charge [22].

On the other hand, rare codons are not uniformly distributed along the mRNA but concentrated in clusters [23], which in many cases are conserved among organisms, suggesting they are under evolutionary pressure [24]. In some cases, these clusters (potentially related to regions of slow translation, as explained above) were associated to particular protein regions, and the proposed explanation for such association was again related to the fine-tuning of the local translation speed and its effects on the folding process [25]. For example, there is a higher frequency of rare codons at the beginning of the coding regions [23], in agreement with the “translational ramp” found by ribosome profiling commented earlier. Similarly, rare codons (as a proxy of slowly translated regions) were also associated to particular secondary structure elements, which have different folding requirements [26, 27]. Rare codons are also in the mRNA regions coding for trans-membrane helices and signal peptides [28], which could be related to the special kinetic requirements of these elements to be properly folded and targeted/inserted into the membrane. Clusters of rare codons were also found downstream the binding sites for the “signal recognition particle” (SRP), which assist protein translocation across membranes [29]. The decrease in translation speed of the nascent polypeptide due to these rare codons (confirmed by ribosome profiling) possibly facilitates SRP recognition and binding. Finally, rare codons were apparently found in domain boundaries [30] so that they could be paying a role as “translational pauses” to facilitate the correct folding of these domains, although such association is controversial and was not found in other works [26]. On the contrary, optimal codons (potentially associated to regions of fast translation) have been found to be associated to aggregation-prone residues [31], perhaps to allow these regions to be rapidly translated and folded before having time for undergoing improper aggregation. The codon usage patterns also point to translation “pauses” between secondary structure elements, as happened with the ribosome occupancy profiles, possibly for allowing these to fold properly.

A recently developed experimental technique (PARS [32]), intended for measuring RNA secondary structure content at a genome-wide scale, also showed that this feature associated to translational speed is related to a number of protein features. For example, the mRNA region around the start codon is enriched in secondary structure [32]. This would be indicative of slower translation at the beginning of the mRNA, in agreement with the observations based on ribosome profiling and codon usage commented before. An increase in mRNA secondary structure has also been reported in the inter-domain and inter-protein linkers in the polycistronic HIV mRNA [33], in agreement with the data commented above based on codon usage.

Hence, although a number of previous works studied the relationship between different mRNA local features related to translational speed and particular local structural aspects of the encoded proteins, a large scale genome-wide study on their relationship with a comprehensive diverse set of annotated functional and structural features was missing. In this work we perform such a large scale study for all mRNAs in two model organisms. We study the relationship between the experimental secondary structure content, ribosomal density, and codon tRNA concentration (as proxies of translational speed) of these mRNAs and the whole repertory of local functional and structural features annotated in Uniprot [34] for the corresponding proteins. We found that many of these protein functional features are indeed reflected in these mRNA properties. One or more proxies of mRNA translation speed have distinctive patterns around the mRNA regions coding for many protein structural and functional features. This suggests that there could be an amino acid-independent mechanism contributing to the coding of important functional features.

## Results and discussion

### mRNA secondary structure and protein features

Table 1 lists the protein features annotated for the Yeast proteome for which it was possible to perform the statistical test described in detail in Methods. Basically, this test compares the distribution of correlations between patterns of mRNA properties (secondary structure and ribosomal occupancy) around a protein feature of interest and a similar distribution constructed with the same number of segments (and of the same length) randomly taken from the pool of mRNAs. If a protein feature is related to a particular mRNA pattern, the first distribution would move away from 0.0 (no correlation) to positive values, while the second distribution is centred at 0.0. The table shows the p-value of the t-test for the null hypothesis that there is no difference between the means of the two distributions. In other words, good (low) p-values can be interpreted as that particular feature having a distinctive pattern of mRNA secondary structure around it, compared with the rest of the proteome.

**Table 1:** Relationship between mRNA and protein features. P-values of the unpaired t-test evaluating the relationship between two mRNA features (ribosome occupancy –E. coli- and secondary structure content –Yeast-) and functional and structural features at the protein level. Missing values are due to a small number of instances of that particular feature in the E. coli or Yeast proteome to perform the test. The number of instances is also indicated for each feature. Uniprot FT features missing in both tests are not included here. P-values lower than the standard 0.05 threshold are highlighted

Feature	Secondary structure		Ribosome occupancy	
Active site	570	3,36E-05	874	2,10E-28
ANCH_R	263	3,12E-03	2091	4,73E-101
Binding site	800	3,01E-14	1365	3,21E-15
Calcium-binding region	16	3,15E-03	-	-
Coiled-coil region	129	1,55E-06	39	7,99E-02
Compositionally biased region	596	2,31E-01	43	2,04E-02
Cross-link	148	1,65E-01	8	6,46E-01
DIS_IUP	870	5,41E-17	13750	3,12E-19
Disulfide bond	86	6,86E-01	99	4,04E-04
DNA-binding region	45	7,69E-01	228	1,81E-24
Domain	890	2,20E-06	1243	2,47E-76
Helix	5789	1,04E-51	13502	3,99E-40
Initiator methionine	272	3,96E-04	342	0,00E + 00
Intramembrane region	-	-	15	2,84E-01
Lipid moiety-binding region	108	8,98E-03	178	7,59E-03
Metal ion-binding site	1046	9,82E-08	2295	7,50E-26
Modified residue	277	2,72E-05	464	4,78E-17
Nucleotide phosphate-binding region	534	2,60E-71	657	0,00E + 00
Peptide	-	-	16	2,73E-12
Propeptide	90	3,77E-01	15	3,99E-02
Region of interest	537	1,26E-06	584	3,10E-04
Repeat	1449	3,36E-08	216	4,10E-03
Short sequence motif	203	5,98E-03	97	1,57E-13
Signal peptide	164	1,83E-33	476	0,00E + 00
Site	127	1,65E-12	256	2,37E-02
Splice variant	16	5,76E-01	10	3,35E-06
Strand	4743	4,48E-33	12282	2,54E-58
Topological domain	2986	4,02E-10	4636	9,24E-22
Transmembrane region	3242	3,22E-31	5704	5,30E-02
Turn	1374	2,42E-20	2951	5,67E-22
Zinc finger region	127	4,61E-01	22	1,62E-05

**Fig. 1:**
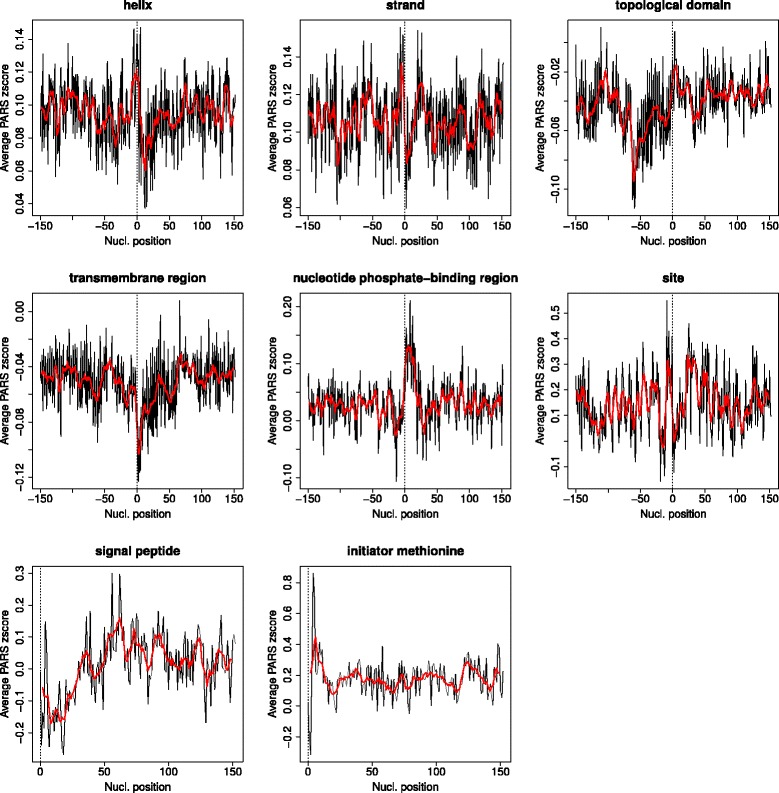
Average patterns of mRNA secondary structure content around a number of protein functional and structural features. “0” indicates the first nucleotide of the region coding for the feature and negative numbers the mRNA region upstream of it (Fig. 1). The red line is a smoothing of the data done with a window of 6 nucleotides. Equivalent plots for all the protein features studied are available in the Additional file 3

It can be seen that the protein features with clearest reflections in the secondary structure of the mRNA are mainly structural features (helix, strand, transmembrane (TM) regions, turns and topological domains). The actual average mRNA secondary structure patterns for some of these protein features are shown in Fig. 1. For protein secondary structure elements, it looks like there is an increase in the mRNA structure just before starting their coding regions followed by a decay, although this pattern is not clear for the turn. For TM regions, a decay in mRNA structure content starts around 50 nucleotides before its beginning and goes down to the beginning itself, where it starts to increase again up to nucleotide ~60 (20 aa, the typical length of a TM helix). The pattern is very similar for “signal peptide”, since in many cases these peptides are indeed TM helices. For topological domain there is an apparent decrease in mRNA secondary structure at the beginning of the corresponding coding region (~50 nucleotides). For “initiator methionine”, which basically indicates the beginning of the protein coding region, there is a very clear peak of secondary structure (slow translation) around the first 20 nucleotides. The functional feature more clearly reflected in the mRNA secondary structure is “nucleotide phosphate-binding region”, for which there is a clear peak of mRNA structure around the first ~25 nucleotides immediately following the starting point of this feature (Fig. 1). This is more or less the length of this feature since, although variable in length as annotated in Uniprot, its average is 7.9 residues (~24 nucleotides). Another functional feature is the very general “site”, used in Uniprot for annotating single residues of interest not covered by other FT keywords. mRNA nucleotides coding for “sites” are also characterized by a particular pattern of secondary structure. Other features do not have a clear reflection at the mRNA secondary structure level according with this test, such as “dna binding”, “disulfide bond”, “zinc-finger region”, etc. (Table 1).

### mRNA ribosome occupancy and protein features

**Fig. 2:**
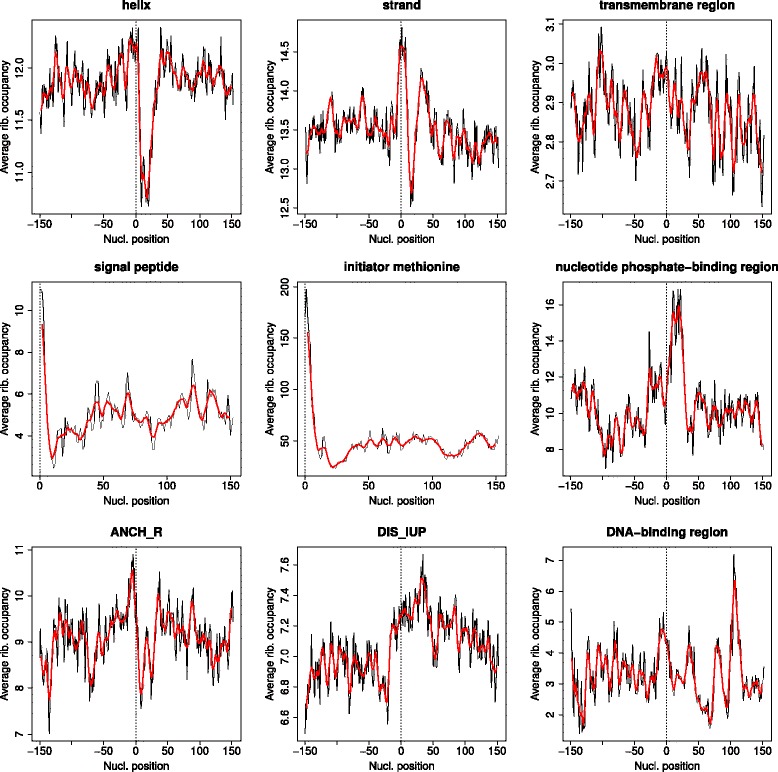
Average patterns of mRNA ribosome occupancy around a number of protein functional and structural features. Same representation as in Fig. 1. Equivalent plots for all the protein features studied are available in the Additional file 3

Table 1 and Fig. 2 show the same results for the ribosomal occupancy, inversely related to the speed of translation. We see again “initiator methionine” and “signal peptides”, with very similar patters to those of mRNA secondary structure (Fig. 2). The pattern is also very similar for “nucleotide phosphate binding region”, with a clear peak of high ribosomal occupancy (slow translation) around the first ~25 nucleotides of the mRNA regions coding for this feature, although in this case the decay of the peak is sharper than for mRNA structure. Both disordered-related features (general disorder –DIS_IUP- and binding related disorder -ANCH_R-) appear as related to mRNA ribosomal occupancy. Again, protein structural features such as “helix”, “domain” or “turn” show up as related to this proxy of mRNA translational speed. While the patterns for “helix” and “strand” are similar to those of mRNA secondary structure, that for TM helix is quite different: whereas it looked like the beginning of the helix was characterized by a local minimum of secondary structure content, it presents a peak of ribosomal occupancy (Fig. 2). A feature apparently related to ribosomal occupancy but not to mRNA structure is “DNA-binding region”, which shows a complex but distinctive pattern of speed of translation (Fig. 2).

### tRNA concentration and protein features

**Fig. 3:**
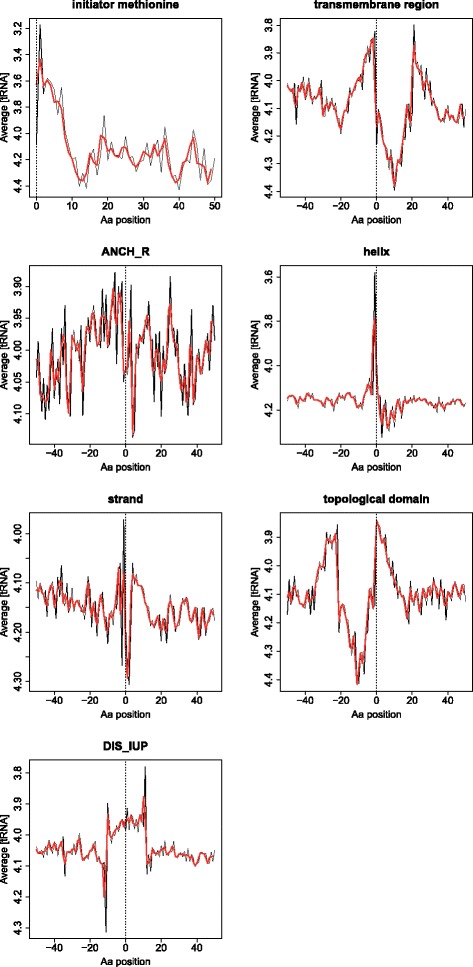
Average patterns of tRNA concentration of the codons around a number of protein functional and structural features. Same representation as in Figs. 2 and 3, except that the X axis now represents codons (residues) instead of nucleotides (although the region covered is the same) and the [tRNA] scale (Y axis) is inverted to maintain the correspondence between high values and (potentially) slow translation, so as to compare with the previous figures. The red line is a smoothing of the data done with a window of 2 residues (6 nucleotides). Equivalent plots for all the protein features studied are available in the Additional file 3

**Fig. 4:**
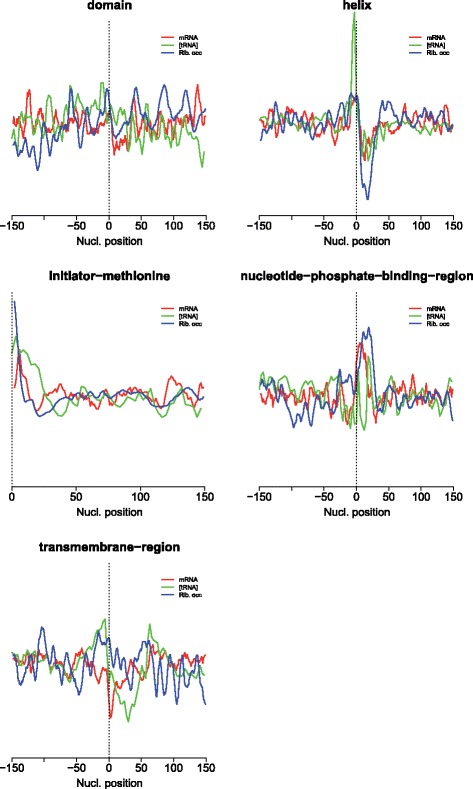
Comparison between the (smoothed) average patterns of mRNA structure, ribosome occupancy and tRNA concentration around a number of protein functional and structural features

In Fig. 3 we show the patterns of tRNA concentration ([tRNA]) for some of the functional features commented above. Although not assessed with a statistical test (see Methods), visually it is clear that some protein functional/structural aspects are also reflected in the patterns of [tRNA] for the corresponding mRNA codons. In this figure we only show features in principle not characterized by conserved amino acids, trying to avoid the problem commented in Methods which precludes the statistical test from being applied, except for the “initiator methionine” (where that bias in principle affects only the first Met). In some cases these patterns are similar to those observed for ribosomal occupancy and mRNA structure (Fig. 4). For example the “translation ramp” observed in the ribosomal occupancy and mRNA secondary structure patterns is also clear here (“initiator methionine”). The pattern for “helix” is also similar in the three proxies of translation speed. In other cases the pattern is different (e.g. disorder –IUPRED-). The high similarity between the [tRNA] pattern for “trans-membrane region” and “topological domain” (also observed for mRNA structure –Fig. 1-) is probably due to the correlation between these two annotations in Uniprot: cytosolic and extracellular domains are separated by trans-membrane helices. Actually, these two patterns are shifted by ~60 nucleotides (20 residues) which is the length of a trans-membrane helix.

For highlighting the similarities these three proxies of mRNA translation speed present in some cases, in Fig. 4 we have drawn the three patterns (smoothed) together for some protein functional features. Note that for “nucleotide phosphate binding”, the [tRNA] pattern is expected to be biased for the reason commented above. A cross-correlation test was applied to the patterns of mRNA secondary structure and ribosome occupancy for these functional features, to quantify their similarity and eventual displacement (Additional file 1). In some cases the two proxies of mRNA translational speed correlated and are in frame (“helix”), while in others they correlate but are displaced, such as for “nucleotide-phosphate-binding”, where they are shifted 10 nucleotides.

To evaluate whether the recently reported relationship between positively charged residues and translational slowdowns [22] could be influencing our results, we calculated the enrichment in this type of amino-acids of the windows around the protein features we explore. In none of them the proportion of positive residues is significantly different from the rest of the proteome, and many even show a depletion in these amino-acid (Additional file 2).

The Additional file 3 contains the patterns of these three mRNA properties for all the protein features studied.

### Examples

**Fig. 5:**
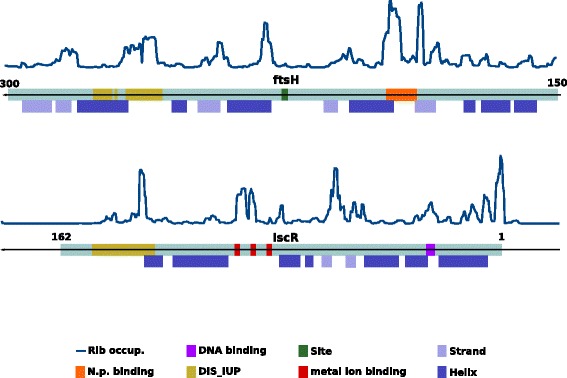
Examples of relationship between mRNA patterns of ribosome occupancy and functional features of the encoded proteins. Upper part: pattern of ribosome occupancy in the 150–300 region of the FtsH gene, where a number of FT features are annotated. Lower part: same representation for the iscR gene. In this case the figure includes the whole gene and hence one of the peaks correspond to the translational ramp at the beginning of the gene. Although not annotated as FT in Uniprot, the region around the only two beta sheets of this protein is also involved in DNA binding. In both cases the proteins are coded in the reverse DNA strand and hence they go from right to left

Figure 5 shows two examples to illustrate the relationship between these mRNA patterns and protein features.

The upper part shows a linear representation of a portion of the FtsH protein of E coli where a number of FT functional and structural features are concentrated. The figure shows the ribosome occupancy pattern of the mRNA coding for that FtsH region. There are three clear regions of high ribosome occupancy which nicely match the three annotated functional features of this protein (“nucleotide-P binding”, “site” and structural disorder (“DIS_IUP”). The “nucleotide-P binding” region is associated to the highest ribosome occupancy. Moreover, for “nucleotide-P binding” and “DIS_IUP” the patterns of these peaks are similar to the average ones commented previously (Fig. 2). Other minor peaks of ribosome occupancy are apparently at the beginning of the β − strands.

The other example is the iscR transcriptional repressor. Again, all the regions of high ribosome occupancy (i.e. slow translation) are associated to functional features. Take into account that the peak at the beginning of the protein (right in the figure) is that of the translational ramp. The peak around the only to beta sheets of this protein is not associated to any FT Uniprot feature. Nevertheless, that region of the protein is also part of the winged “helix-turn-helix” (wHTH) DNA binding motif (of which only the HTH part is annotated in Uniprot as “DNA_binding”).

### Discussion

The degeneracy of the genetic code makes it possible to code the same chain of amino acids with many different mRNA sequences. In the other hand, the nucleotide sequence of the mRNA determines some properties related to its local translational efficiency, which in turn could have an effect on the structure and function of the encoded polypeptide. It is reasonable to think that the evolution could be playing with these semi-independent features (amino acid type and translation speed) to obtain the final structure and function of the protein. Nevertheless, it is clear that the amino acid types are by far the main determinants of the protein features. The translation speed profile would be playing a very subtle effect, to fine-tune some minor details of certain proteins, or to help/reinforce the main features dictated by the amino acid composition. Indeed, our results show that the local variations in mRNA features around structural/functional regions are very subtle (e.g. low z-scores), and the dispersion observed between the individual cases is very high. Moreover, these two codes are probably entangled and not fully independent. Indeed, direct relationships between particular amino-acid compositions and translational speeds were found [22, 35].

From the early works reporting a relationship between synonymous codon usage and overall translational efficiencies of the proteins, new methodological developments allowed to start studying this phenomenon at the local level. These methodological developments, which allow to experimentally determine mRNA local characteristics at a genomic scale and at 1-nucleotide resolution, allowed us to perform the first large scale analysis on the relationship between local patterns of mRNA proxies of translation speed and protein functional features.

Our analysis of three proxies of mRNA translation speed shows that this is related to many structural and functional features at the protein level. In some cases, these three experimentally-obtained mRNA features (mRNA structure, ribosome occupancy and tRNA concentration) follow similar patterns, what is interesting specially taking into account that they were obtained in organisms as different as a prokaryote (E. coli) and an eukaryote (Yeast). A possible explanation for these similarities could be that, since the main force dictating the nucleotide composition is the requirement for amino-acids, and the effects of these others is very subtle, the evolution can be playing with all of them at the same time to obtain the required slowing down in translation. I.e. in some cases it will be possible to play with the codon usage to obtain a slowing down (rare tRNAs) while in others this is not possible (i.e. amino-acid with only 1 codon) and it is the secondary structure what has to be changed (by changing other regions of the mRNA to change the pairings). So, a mixture of instances where the slow downs are achieved by one mechanism or another could result in similar average patterns. Also, it is important to remember that these three mRNA features are not totally independent (e.g. higher secondary structure = > lower speed = > higher ribosome occupancy) which can also contribute to these similarities

Our results are in agreement with those recently obtained by Yang et al. [36], who found an overall inverse relationship between the evolutionary conservation of the codons and their translational speed. The evolutionary conservation of the codons can be due to the functional importance of the residues they code, among other things such as residue structural importance or mRNA-related importance.

We found the “translational ramp” also reported by other authors at the beginning of the coding regions. We also saw a general trend of translation pausing at the beginning of protein secondary structure elements, in agreement with that previously reported by other authors. Not only purely structural features are reflected in these mRNA patterns, but also functional ones. The more intriguing of these is “nucleotide-phosphate binding”, which present a pattern of “translational deceleration” covering more or less the (average) length of the mRNA region coding for it, and which is evident in the three proxies of elongation speed. This prompts us to investigate further in the future the relationship between mRNA features and more specific functional features (binding of large/charged ligands, active sites, protein binding sites, etc.) after this first exploratory “bird-view” work with the very general Uniprot “FT” features. The recent finding that elongation speed (inferred from mRNA structure) is slowed down at evolutionary conserved sites [36] would be in agreement with our results due to the known relationship between functional sites and sequence conservation.

Our results are in agreement with the hypothesis generally used for explaining the effect of mRNA features on the structure of the protein. Assuming that the nascent protein chain starts to fold as it gets out from the ribosome (co-translational folding), a local fine-tune of the translation speed would allow, for example, to slow down the elongation of a certain region to give time for a ligand to be inserted before this region gets folded (e.g. the nucleotide in the example above), without compromising other regions which have to be translated faster.

## Conclusions

Our results support the idea that the mRNA is coding for more than mere chains of amino acids, thanks to the degeneracy of the genetic code. It looks like genes code proteins’ functional features using overlapping (and probably entangled) messages of different nature. Apart from a better understanding of the relationship between protein function and its codification at the genomic level, the found relationships between mRNA features and protein structural/functional characteristics could help in devising new ways of engineering subtle changes in protein function. While changing amino acids either has a drastic effect on the protein or no effect at all, playing with mRNA features could serve as a way of fine-tuning protein function. These results could have also practical implications for the heterologous expression of proteins: the general strategy currently employed for heterologously expressing a protein is to use abundant codons of the host organism, aiming at globally increasing the translational efficiency. This approach is known to fail in many cases, generating non-functional or aggregating proteins. Even “optimizing” a gene in its same organism by using optimal codons can produce unexpected results such as altered structure or function [37], decreased protein production and/or activity, and negative effects on the overall fitness of the organism [18]. In some cases the reason could be that this change in the codon pattern destroys these mRNA local profiles related to the structure and function of the protein. According with our results, the inserted gene should be designed trying to respect these patterns (adapting them to the tRNA pool of the host organism). Although the patterns found so far are in general very subtle, for particular cases with very clear patterns these could be used as a way of predicting protein functional sites using single gene sequences (almost all current methods for predicting functional sites from sequence information require many sequences of the same family to work).

## Methods

**Fig. 6:**
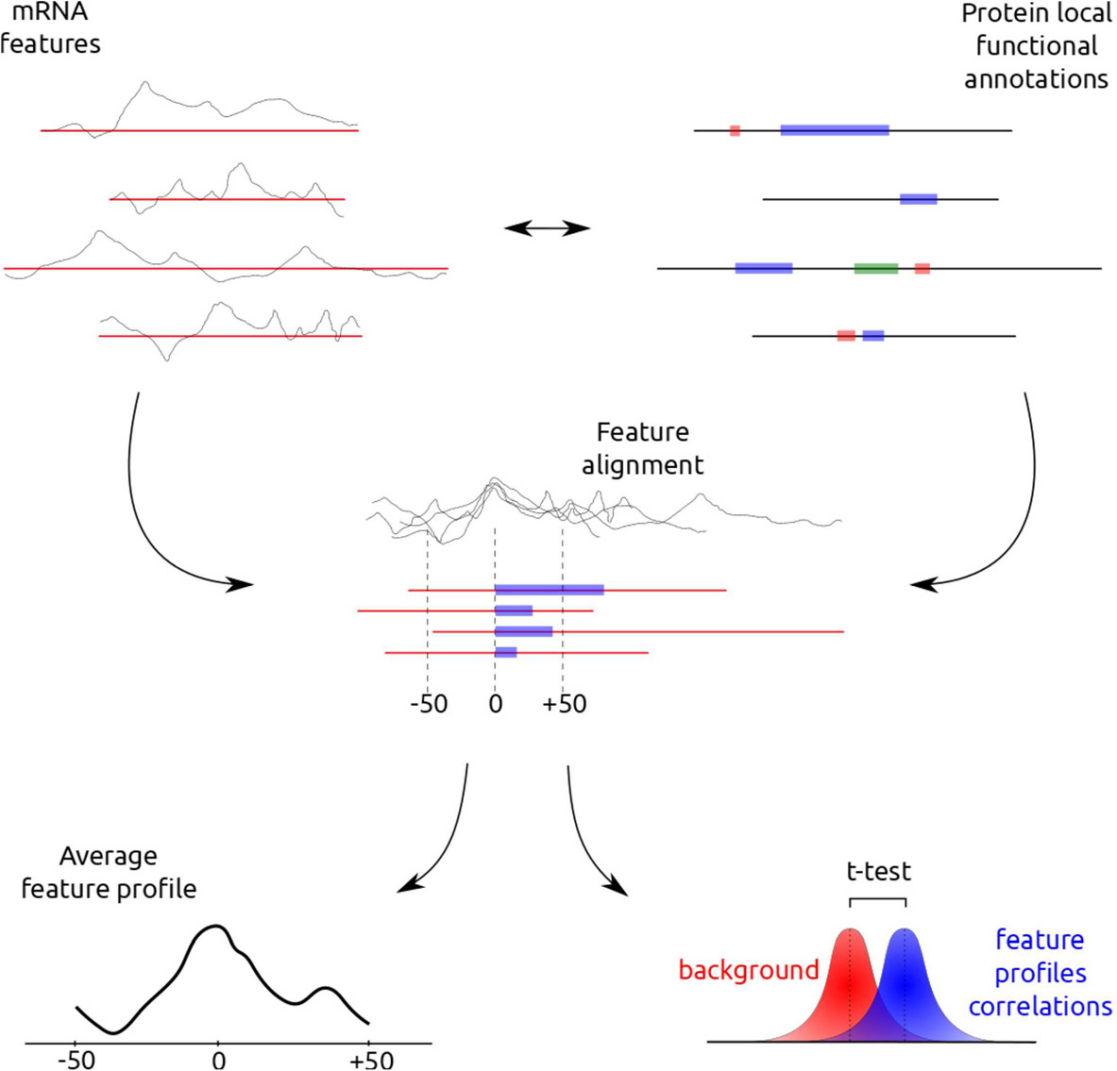
Schema of the methodology used for studying the relationship between mRNA local features and functional/structural features of the encoded proteins. For a given Uniprot FT feature (purple), all its instances in the proteome are aligned respect to the first residue. This determines an equivalent alignment of the corresponding mRNA coding regions (red lines) and their associated feature profiles (secondary structure, ribosome occupancy or tRNA concentration –black curves-). These profiles are averaged and visualized to represent the behaviour of a particular mRNA property in the neighbourhood of that FT feature (bottom left). Additionally, a distribution with the values of pair-wise correlations between these profiles is generated and compared with an equivalent distribution generated from profiles of the same size randomly taken from the proteome (bottom right)

The procedure followed for evaluating the relationship between mRNA features and functional regions of the encoded proteins is depicted in Fig. 6. Experimental information at the nucleotide level on ribosomal occupancy (a proxy for speed of translation) and tRNA concentration for E. coli, and mRNA secondary structure (for Yeast), were combined with the functional features annotated in Uniprot for the corresponding residues of the encoded proteins so as to assess which functional features were “reflected” in the mRNA and how.

The ribosomal profiling data generated by Li et al. [21] was retrieved from GEO [38] (GEO:GSM872393). This data, obtained by deep sequencing of a ribosome protected mRNA sample, consist in a value (in arbitrary units) for each mRNA nucleotide of E. coli. Such value quantifies the ribosomal density at that particular site and is inversely correlated with the speed of translation of that part of the mRNA, since a region of slow translation would statistically present more ribosomes stalled in its instances in the sequenced sample. A very small proportion of nucleotides present occupancy values many orders of magnitude higher that the average which are probably errors. In order to remove these “outliers”, we exclude values 1.5•σ higher than the average. On the other hand, the Uniprot [39] entries for E. coli K12 proteins were retrieved (4303 proteins). These entries include information on diverse features of the protein (“FT” features), together with the range of residues these features are associated to. The vocabulary used in the FT descriptions is very general and includes a broad range of functional (e.g. “DNA binding”) as well as structural (e.g. “helix”) features, experimentally determined or predicted. These FT annotations are very suitable for this study, which looks for relationships between mRNA characteristics and features of the proteins in the broadest sense, without a-priory restricting to a particular type of feature. Moreover, each FT feature is studied separately and they are never mixed. We added two additional features to that set of FT annotations: disordered regions predicted by IUPRED [40], and disordered regions potentially involved in protein interactions as predicted by ANCHOR [41]. In both cases, we take the protein residues reported by these two programs using their default parameters.

To study the ribosome occupancy profile in the neighbourhood of a given functional feature we start by aligning all the instances of that feature in the proteome at their starting residues (Fig. 6). This is because some FT features are instantiated in segments of different length (e.g. binding sites of different sizes). This alignment determines that of the mRNA regions coding for these protein regions associated to that particular feature (Fig. 6). Consequently, we can average the ribosome occupancy value for each nucleotide position in this alignment: i.e. the average value for the 1st nucleotide of the 1st residue of that feature, the 2nd nucleotide of the 1st residue, etc. We explore a window of 150 nucleotides around the starting point of the features (Fig. 6).

We performed a similar analysis for the mRNA secondary structure. In this case, we took the data generated by Kertsz et al. [32], who used a experimental approach based on deep sequencing for determining the degree of pairing of Yeast’s RNAs at the single nucleotide resolution. In this approach, an mRNA sample is treated in parallel with two different RNases, that cut preferentially in doubled and single stranded regions respectively. Deep sequencing of the resulting fragments generates, for each RNase, a cleavage profile along the mRNA sequence. A log-ratio of both profiles (PARS score) provides a quantification, for each nucleotide, of its propensity to be paired. The higher the value, the higher the proportion of that nucleotide to be paired within the population of that RNA species. From the original Kertsz et al. data, we filtered out RNA species different from mRNA. We also removed from the mRNA the regions not directly coding for proteins, such as the 5’ and 3’ UTRs. Since the ranges of PARS score for each of the 4 nucleotides are very different, we normalize them by transforming to z-scores. Among other things, this avoids biases introduced by the particular amino acids found at the functional sites. For example, a functional feature characterized by (conserved) phenylalanines (F) would be associated to a PARS value biased towards that of the uracyl (U), since the two codons for F are UUU and UUC. Note that such normalization was not done for the ribosome occupancy data (see above) since there were no significant differences in the distributions of values for the 4 nucleotides (data not shown). We retrieved from Uniprot the Yeast proteome (7795 entries) containing the FT annotations, as previously done for E. coli. We also included IUPRED and ANCHOR predictions as additional features. We aligned these Uniprot sequences with the amino acid sequences in Kertsz’s data and excluded 9 cases with more than 5 % discrepancies (after taking into account those sequence discrepancies reported in Uniprot itself). We followed the same procedure previously described for E. coli for studying the mRNA secondary structure profile in the neighbourhood of a given protein feature (Fig. 6).

In both cases, we performed a correlation test to statistically assess whether a given protein functional feature has a distinctive pattern of a particular mRNA property around it, significantly different from the average of the coding regions. For that, we took all the vectors of values of a given mRNA property (z-scores of PARS –secondary structure- and ribosome occupancy) in the −50/+50 nucleotide neighbourhood of all the instances of a given functional feature, and calculated their pair-wise Pearson’s correlations. This gives us a distribution of correlations for that particular feature and that particular mRNA property (Fig. 6). We generated an equivalent distribution with 1000 vectors of the same length randomly taken from the pool of mRNAs. If a functional feature is related to a distinctive pattern of mRNA property (as illustrated in Fig. 6) the distribution of correlation values will be shifted to high values, whereas the equivalent distribution for the mRNA segments randomly taken will be centred around 0.0. Consequently, we performed a one side unpaired t-test to evaluate whether the mean correlation is larger in the first case. For 32 FT features there were enough instances in the proteome of the corresponding organism to perform this statistical test (Table 1).

Additionally, we took from [42] the experimental data on the relative concentration of the tRNA species in E. coli: fraction of a given tRNA respect to the total amount of tRNA for a growth rate of 0.4 doubling per hour. We use this data to generate profiles similar to those described above for ribosomal occupancy and mRNA structure around the protein functional/structural features. We include those for illustrating how some protein functional aspects are also apparently reflected in another mRNA feature potentially related to translation speed. Nevertheless, in this case it is difficult to devise a statistical test as that applied for ribosome occupancy and mRNA structure since the tRNA concentration is highly affected by the amino acid type, especially for amino acids represented by one or two codons in the genetic code. For example, Cys, a common amino acid in active sites, is represented by only two codons. Consequently, the [tRNA] value for the mRNA codons coding for Cys will be constrained by these values. The generation of the background distribution of correlations (Fig. 6) would have to take that into account, which is not trivial.

## Additional files

Additional file 1:
**Enrichment of positively charged residues in the regions around protein functional features analyzed.**
Additional file 2:
**Patterns of mRNA secondary structure, ribosome occupancy and [tRNA] for all the protein features analyzed.**
Additional file 3:
**Cross-correlation test to quantify the similarity as well as the eventual shift of the secondary structure and ribosome occupancy patterns of Fig.** **4** **.**

## References

[1] Hurst LD (2011). Molecular genetics: The sound of silence. Nature.

[2] Sauna ZE, Kimchi-Sarfaty C (2011). Understanding the contribution of synonymous mutations to human disease. Nat Rev Genet.

[3] Brest P, Lapaquette P, Souidi M, Lebrigand K, Cesaro A, Vouret-Craviari V (2011). A synonymous variant in IRGM alters a binding site for miR-196 and causes deregulation of IRGM-dependent xenophagy in Crohn's disease. Nat Genet.

[4] Chursov A, Walter MC, Schmidt T, Mironov A, Shneider A, Frishman D (2012). Sequence-structure relationships in yeast mRNAs. Nucleic Acids Res.

[5] Kudla G, Murray AW, Tollervey D, Plotkin JB (2009). Coding-sequence determinants of gene expression in Escherichia coli. Science.

[6] Tuller T, Carmi A, Vestsigian K, Navon S, Dorfan Y, Zaborske J (2010). An evolutionarily conserved mechanism for controlling the efficiency of protein translation. Cell.

[7] Duret L (2000). tRNA gene number and codon usage in the C. elegans genome are co-adapted for optimal translation of highly expressed genes. Trends In Genetics.

[8] Ikemura T (1985). Codon usage and tRNA content in unicellular and multicellular organisms. Mol Biol Evol.

[9] Moriyama EN, Powell JR (1997). Codon usage bias and tRNA abundance in Drosophila. J Mol Evol.

[10] Percudani R, Pavesi A, Ottonello S (1997). Transfer RNA gene redundancy and translational selection in Saccharomyces cerevisiae. J Mol Biol.

[11] Akashi H, Eyre-Walker A (1998). Translational selection and molecular evolution. Curr Opin Genet Dev.

[12] Ernst J (1988). Codon usage and gene expression. Trends Biotechnol.

[13] Frenkel-Morgenstern M, Danon T, Christian T, Igarashi T, Cohen L, Hou Y-M, Jensen LJ (2012). Genes adopt non-optimal codon usage to generate cell cycle-dependent oscillations in protein levels. Mol Syst Biol.

[14] Wohlgemuth SE, Gorochowski TE, Roubos JA (2013). Translational sensitivity of the Escherichia coli genome to fluctuating tRNA availability. Nucleic Acids Res.

[15] Fredrick K, Ibba M (2010). How the sequence of a gene can tune its translation. Cell.

[16] Kolb VA (2001). Cotranslational protein folding. Mol Biol.

[17] Kimchi-Sarfaty C, Oh JM, Kim I-W, Sauna ZE, Calcagno AM, Ambudkar SV (2007). A "silent" polymorphism in the MDR1 gene changes substrate specificity. Science.

[18] Agashe D, Martinez-Gomez NC, Drummond DA, Marx CJ (2012). Good codons, bad transcript: large reductions in gene expression and fitness arising from synonymous mutations in a key enzyme. J Mol Evol.

[19] Ingolia NT, Ghaemmaghami S, Newman JRS, Weissman JS (2009). Genome-wide analysis in vivo of translation with nucleotide resolution using ribosome profiling. Science.

[20] Dana A, Tuller T (2012). Determinants of Translation Elongation Speed and Ribosomal Profiling Biases in Mouse Embryonic Stem Cells. PLoS Comput Biol.

[21] Li G-W, Oh E, Weissman JS (2012). The anti-Shine-Dalgarno sequence drives translational pausing and codon choice in bacteria. Nature.

[22] Charneski CA, Hurst LD (2013). Positively charged residues are the major determinants of ribosomal velocity. PLoS Biol.

[23] Phoenix DA, Korotkov E (1997). Evidence of rare codon clusters within Escherichia coli coding regions. FEMS Microbiol Lett.

[24] Clarke TF, Clark PL (2008). Rare codons cluster. PLoS One.

[25] Chartier M, Gaudreault F, Najmanovich R (2012). Large scale analysis of conserved rare codon clusters suggests an involvement in co-translational molecular recognition events. Bioinformatics.

[26] Saunders R, Deane CM (2010). Synonymous codon usage influences the local protein structure observed. Nucleic Acids Res.

[27] Ta T, Argos P (1996). Protein secondary structural types are differentially coded on messenger RNA. Protein Sci.

[28] Power PM, Jones RA, Beacham IR, Bucholtz C, Jennings MP (2004). Whole genome analysis reveals a high incidence of non-optimal codons in secretory signal sequences of Escherichia coli. Biochem Biophys Res Commun.

[29] Pechmann S, Chartron JW, Frydman J (2014). Local slowdown of translation by nonoptimal codons promotes nascent-chain recognition by SRP in vivo. Nat Struct Mol Biol.

[30] Ta T, Argos P (1996). Ribosome-mediated translational pause and protein domain organization. Protein Sci.

[31] Lee Y, Zhou T, Tartaglia GG, Vendruscolo M, Wilke CO (2010). Translationally optimal codons associate with aggregation-prone sites in proteins. Proteomics.

[32] Kertesz M, Wan Y, Mazor E, Rinn JL, Nutter RC, Chang HY (2010). Genome-wide measurement of RNA secondary structure in yeast. Nature.

[33] Watts JM, Dang KK, Gorelick RJ, Leonard CW, Bess JW, Swanstrom R (2009). Architecture and secondary structure of an entire HIV-1 RNA genome. Nature.

[34] UniProt_Consortium (2010). Ongoing and future developments at the Universal Protein Resource. Nucleic Acids Res.

[35] Gardin J, Yeasmin R, Yurovsky A, Cai Y, Skiena S, Futcher B. Measurement of average decoding rates of the 61 sense codons in vivo. Elife. 2014;3. 10.7554/eLife.03735 PMC4371865 25347064

[36] Yang J-R, Chen X, Zhang J (2014). Codon-by-Codon Modulation of Translational Speed and Accuracy Via mRNA Folding. PLoS Biol.

[37] Zhou M, Guo J, Cha J, Chae M, Chen S, Barral JM (2013). Non-optimal codon usage affects expression, structure and function of clock protein FRQ. Nature.

[38] Barrett T, Troup DB, Wilhite SE, Ledoux P, Evangelista C, Kim IF (2011). NCBI GEO: archive for functional genomics data sets—10 years on. Nucl Acids Res.

[39] Consortium U (2009). The Universal Protein Resource (UniProt) 2009. Nucleic Acids Res.

[40] Dosztanyi Z, Csizmok V, Tompa P, Simon I (2005). IUPred: web server for the prediction of intrinsically unstructured regions of proteins based on estimated energy content. Bioinformatics.

[41] Dosztanyi Z, Meszaros B, Simon I (2009). ANCHOR: web server for predicting protein binding regions in disordered proteins. Bioinformatics.

[42] Dong H, Nilsson L, Kurland CG (1996). Co-variation of tRNA abundance and codon usage in Escherichia coli at different growth rates. J Mol Biol.

