# Supplementary material for: Protein functional features are reflected in the patterns of mRNA translation speed
Source: BMC Genomics. 2015 Jul 9;16(1):513. doi: 10.1186/s12864-015-1734-7 (PMC4497413; doi:10.1186/s12864-015-1734-7)

Amino-acid independent protein functional features coded in the mRNA

Daniel López & Florencio Pazos

Supplementary File 3

Cross-correlation between the mRNA secondary structure and ribosome occupancy patterns for the protein functional features discussed in Figure 4. The plots show the correlation obtained (Y-axis) when one of the patterns is displaced a given number of positions (X-axis) respect to the other.

Domain

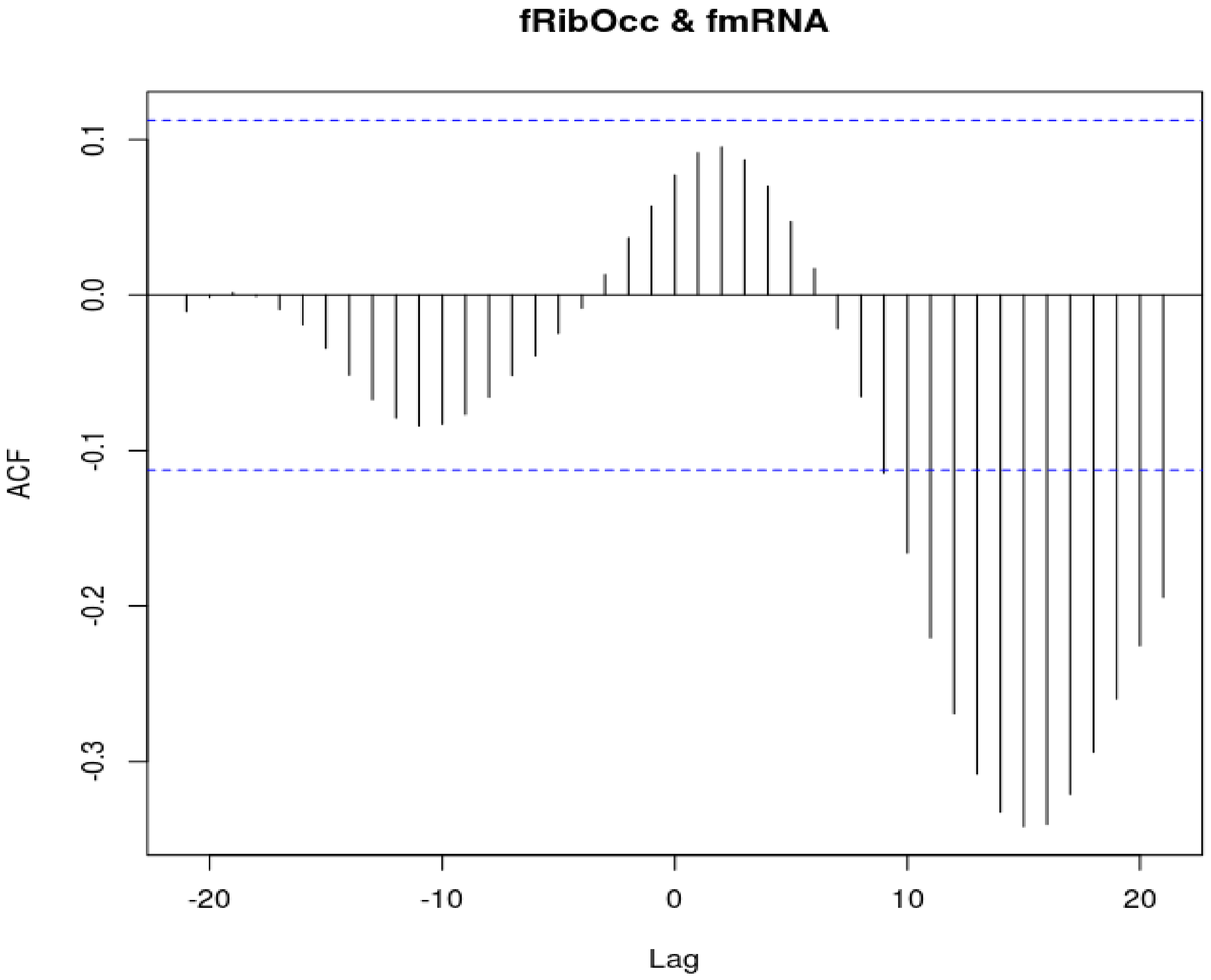

helix

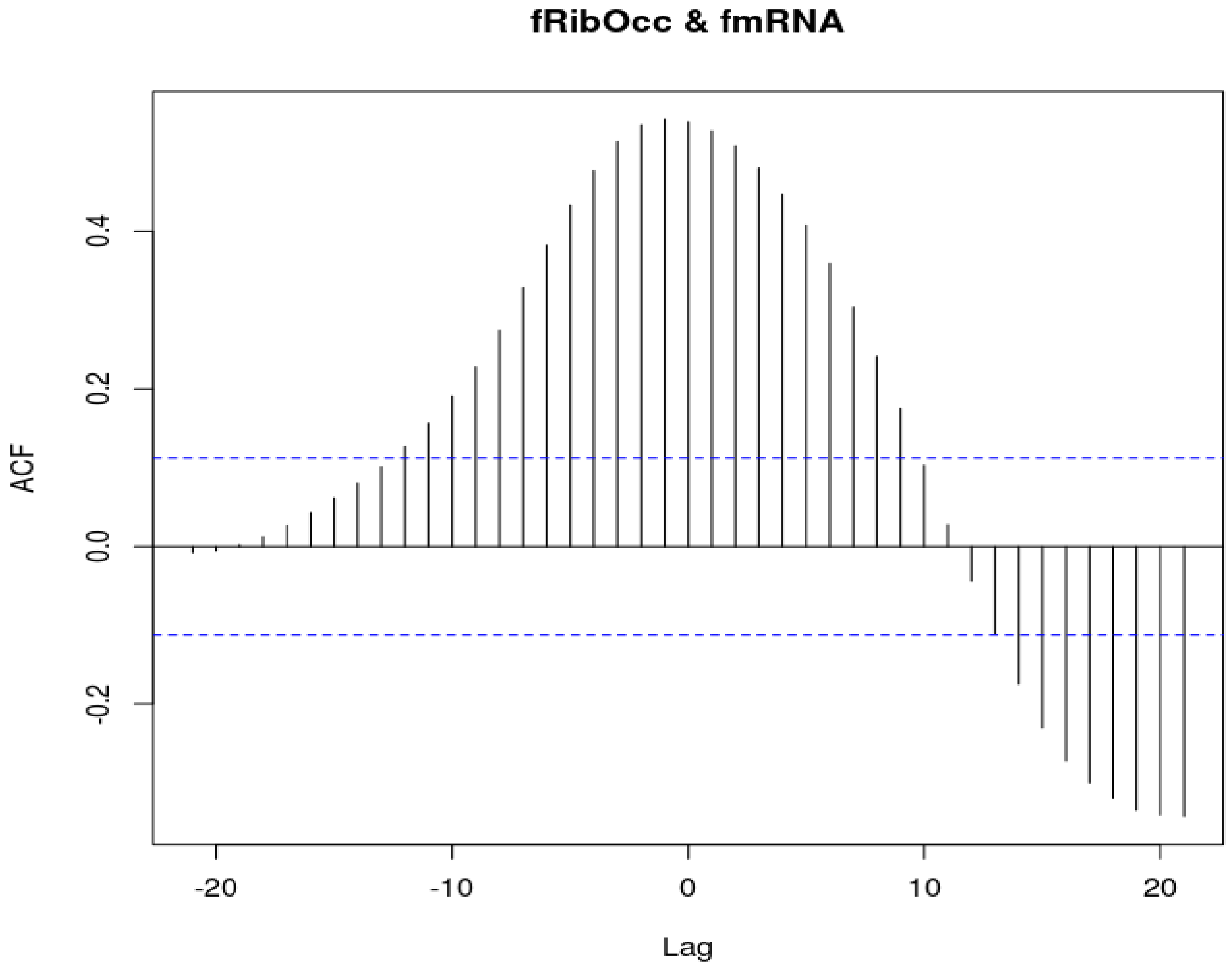

initiator-metionine

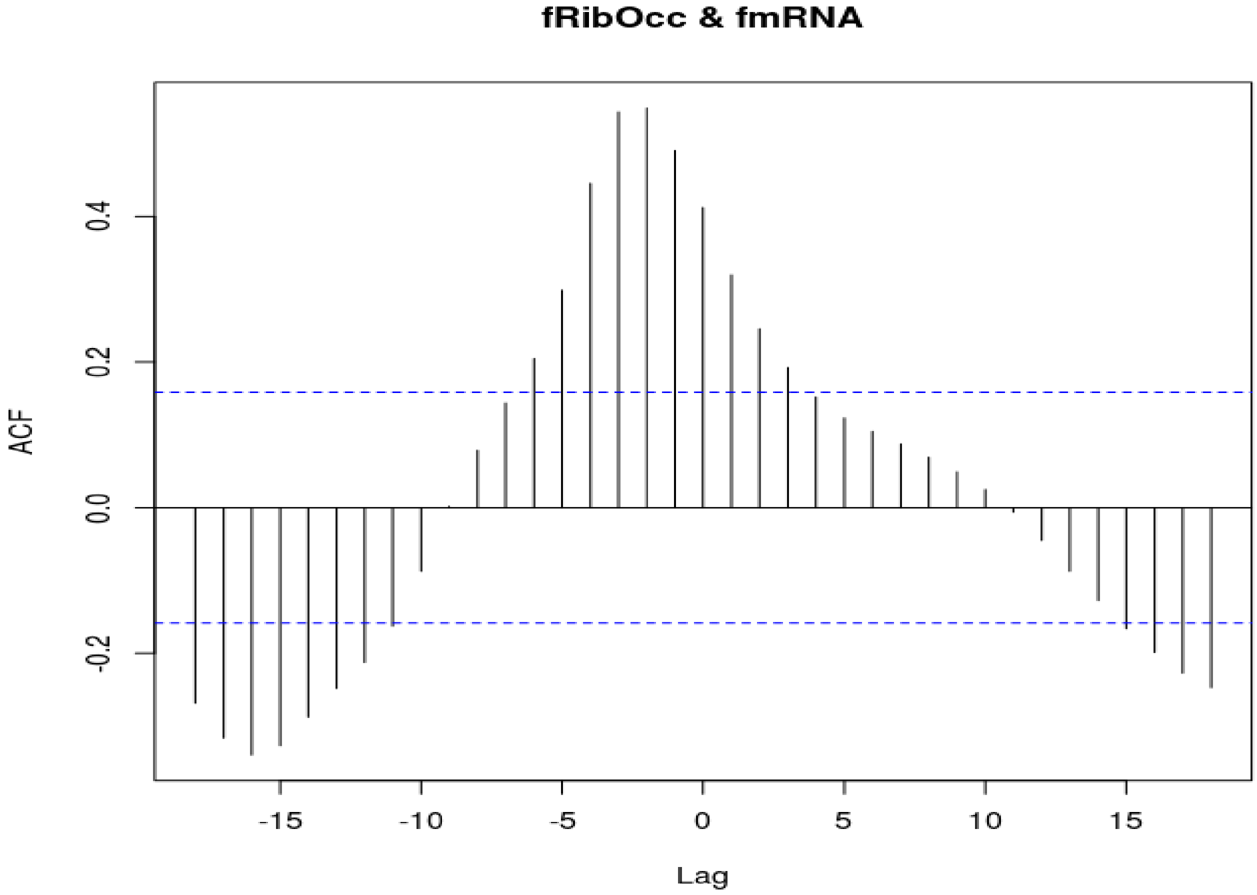

nucleotide-phosphate-binding

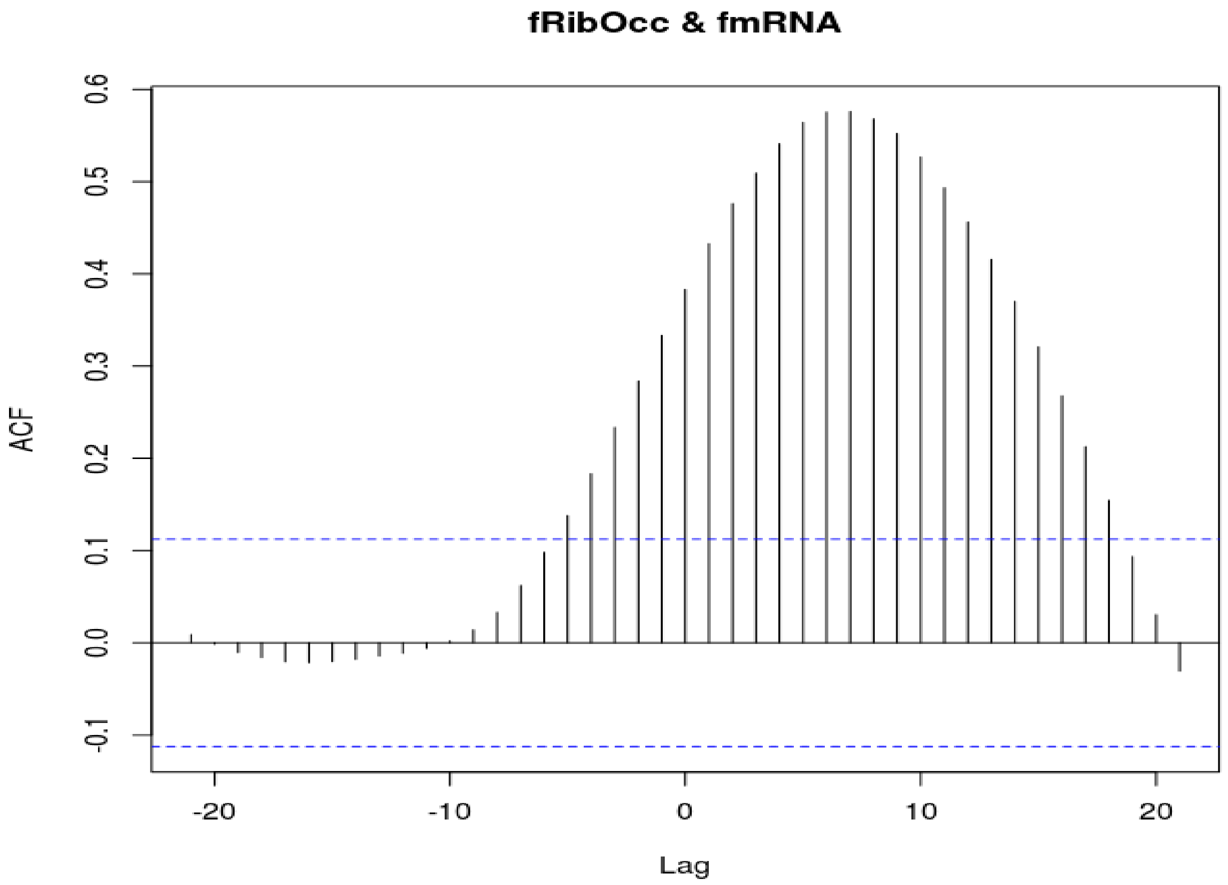

Transmembrane region

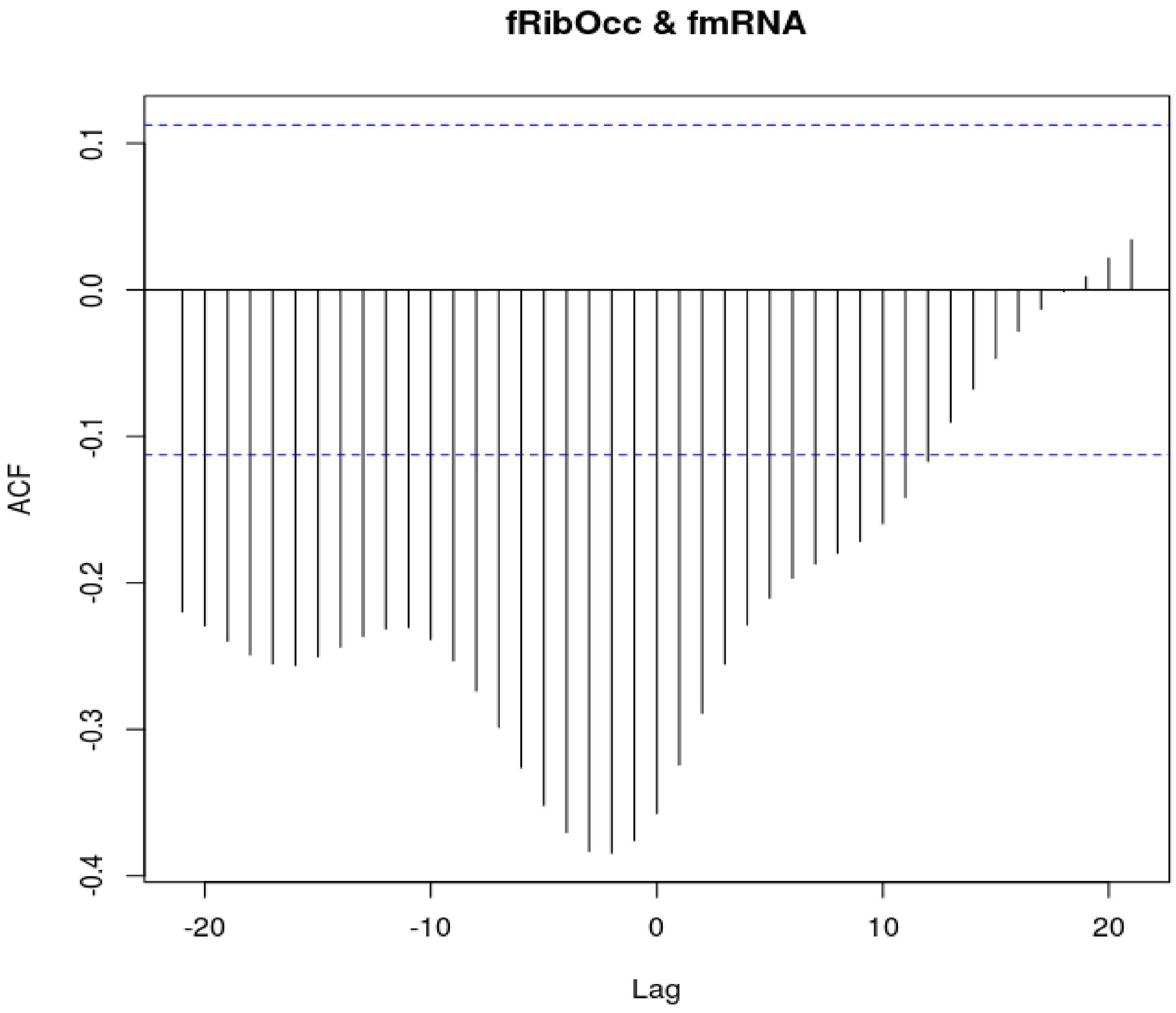

Supplement: Additional file 1: — Enrichment of positively charged residues in the regions around protein functional features analyzed. [file 12864_2015_1734_MOESM1_ESM.pdf]
