# Supplementary material for: Protein functional features are reflected in the patterns of mRNA translation speed
Source: BMC Genomics. 2015 Jul 9;16(1):513. doi: 10.1186/s12864-015-1734-7 (PMC4497413; doi:10.1186/s12864-015-1734-7)
Supplement: Additional file 2: — Patterns of mRNA secondary structure, ribosome occupancy and [tRNA] for all the protein features analyzed. [file 12864_2015_1734_MOESM2_ESM.pdf]

Amino-acid independent protein functional features coded in the mRNA

Daniel López & Florencio Pazos

Supplementary File 1

Enrichment in positively charged residues of the windows around functional features

We calculate the frequency of the 20 aminoacids in the whole proteome of E coli

Amino-acid	Total (n)	Total (%)
A	131793	9,489
C	16156	1,163
D	71537	5,151
E	80014	5,761
F	54014	3,889
G	102292	7,365
H	31437	2,263
I	83340	6,000
K	61343	4,417
L	147764	10,639
M	39166	2,820
N	55062	3,964
P	61297	4,413
Q	61671	4,440
R	76947	5,540
S	81093	5,839
T	75044	5,403
U	3	0,000
V	98044	7,059
W	21283	1,532
X	15	0,001
Y	39593	2,851

These values are very similar to those extracted for whole databases such as Uniprot.

For a given feature, we calculate the frequencies (f) of positively charged residues (Arg and Lys) in the windows around these featres (see Methods) and the z-score of these values respect to the distribution of frequencies of Arg+Lys in the proteome of E coli: $z=(f-\mu)/\sigma$, where $\mu=0.10325$ and $\sigma=0.03343476$. A positive and large z-score (a typical threshold is 2.0) would indicate an enrichment of positive residues in the windows surrounding a particular feature.

The results show that none of the features is specially enriched in positive residues, being “topological domain” that with the highest proportion of charged residues (z=1.4). Many of the features even present some depletion in positive residues (negative z-scores), e.g. “DNA-binding”, “nucleotide-P-binding”.

feature	frequency	Number of samples	Z-score
active-site	0,0971659	874	0,3851081928
ANCH_R	0,114529	2091	0,4828806906
binding-site	0,100513	1365	0,0794382852
calcium-binding-region	0,108911	2	-0,0832666363
coiled-coil-region	0,119395	39	0,0190221195
compositionally-biased-region	0,103886	43	-1,146100645
cross-link	0,0870827	8	0,4199521695
DIS_IUP	0,107988	13750	-0,4362675252
disulfide-bond	0,0933014	99	0,004127441
DNA-binding-region	0,133823	228	-0,159420914
domain	0,105662	1243	-0,4835476612
helix	0,103354	13502	0,1031262076
initiator-methionine	0,116126	342	-0,1819693038
intramembrane-region	0,0660066	15	-0,0363095174
lipid-moiety-binding-region	0,0971863	178	-0,0818609136
metal-ion-binding-site	0,0979198	2295	-0,1813591603
modified-residue	0,109334	464	-1,1948313671
non-standard-amino-acid	0,0726073	3	-0,208217436
ucleotide-phosphate-binding-regio	0,100466	657	-0,9164922972
peptide	0,105906	16	0,3260080228
propeptide	0,11415	15	0,9144076404
region-of-interest	0,103416	584	0,0721404909
repeat	0,117291	216	0,1417088084
short-sequence-motif	0,106698	97	0,1693148089
signal-peptide	0,0886635	476	-1,1139125868
site	0,0962883	256	-0,2975526069
splice-variant	0,149606	10	0,3373435311
strand	0,102036	12282	0,6871292033
topological-domain	0,0649304	4636	1,3864612756
transmembrane-region	0,0633011	5704	0,0049648928
turn	0,103388	2951	0,0031105353
zinc-finger-region	0,126224	22	0,1819663129
