# Supplementary material for: Protein functional features are reflected in the patterns of mRNA translation speed
Source: BMC Genomics. 2015 Jul 9;16(1):513. doi: 10.1186/s12864-015-1734-7 (PMC4497413; doi:10.1186/s12864-015-1734-7)
Supplement: Additional file 3: — Cross-correlation test to quantify the similarity as well as the eventual shift of the secondary structure and ribosome occupancy patterns of Fig. 4 . [file 12864_2015_1734_MOESM3_ESM.pdf]

Amino-acid independent protein functional features coded in the mRNA

Daniel López & Florencio Pazos

Supplementary File 2

Average patterns of the three mRNA features explored (ribosome occupancy, secondary structure and [tRNA]) for all protein features evaluated.

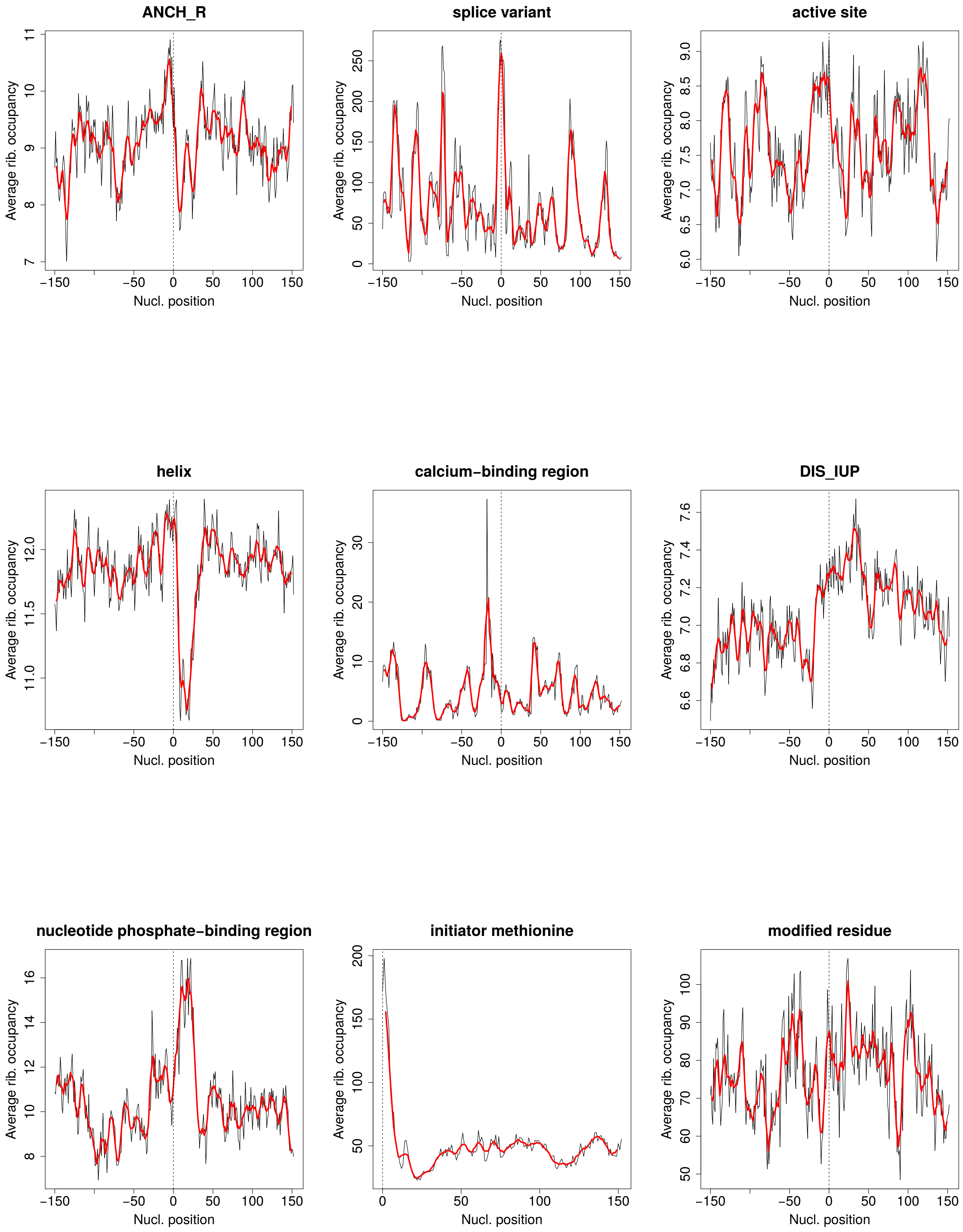

ANCH_R
Average rib. occupancy
Nucl. position
splice variant
Average rib. occupancy
Nucl. position
active site
Average rib. occupancy
Nucl. position
helix
Average rib. occupancy
Nucl. position
calcium−binding region
Average rib. occupancy
Nucl. position
DIS_IUP
Average rib. occupancy
Nucl. position
nucleotide phosphate−binding region
Average rib. occupancy
Nucl. position
initiator methionine
Average rib. occupancy
Nucl. position
modified residue
Average rib. occupancy
Nucl. position

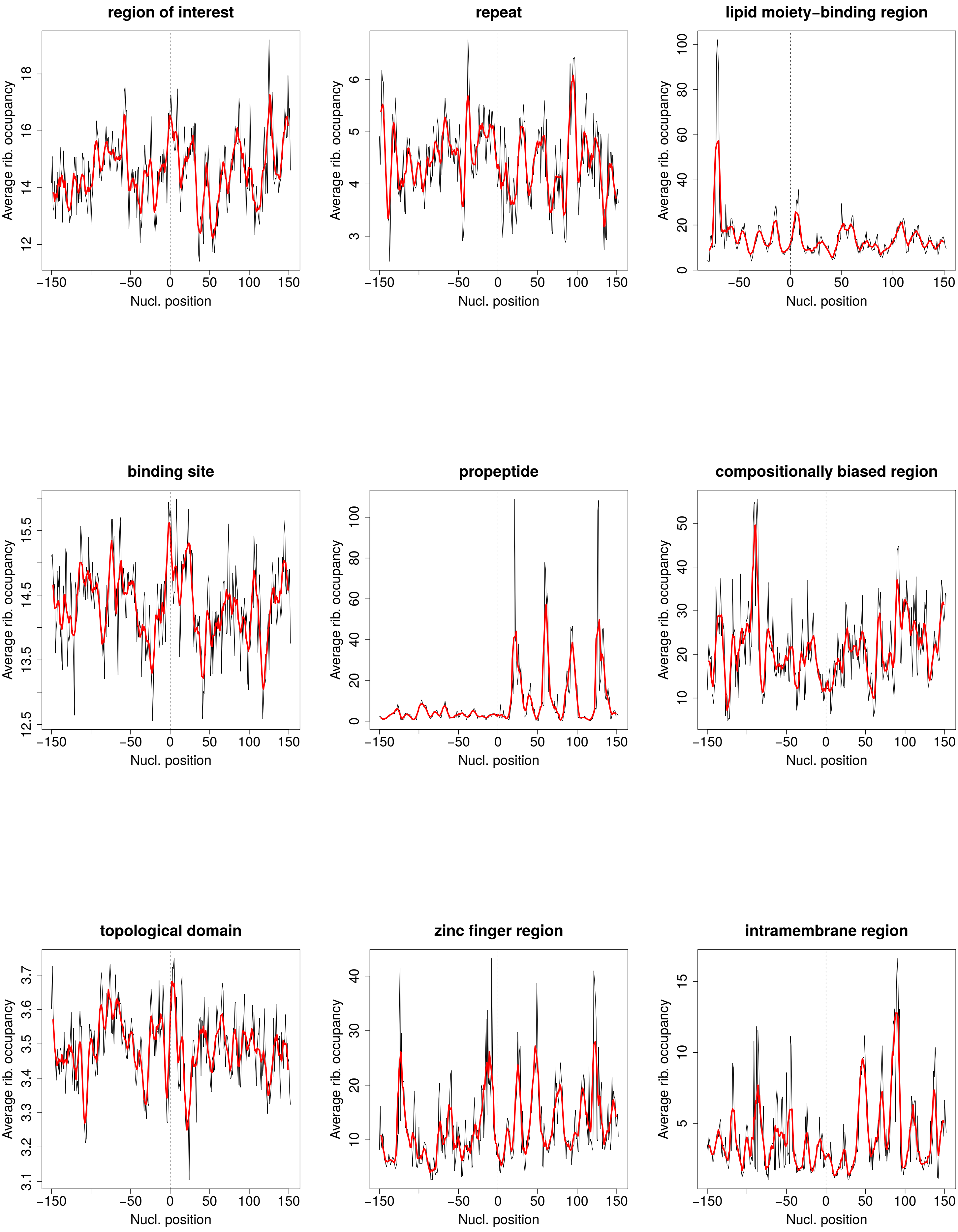

region of interest
repeat
lipid moiety−binding region
binding site
propeptide
compositionally biased region
topological domain
zinc finger region
intramembrane region
Average rib. occupancy
Nucl. position
−150
−50
0
50
100
150

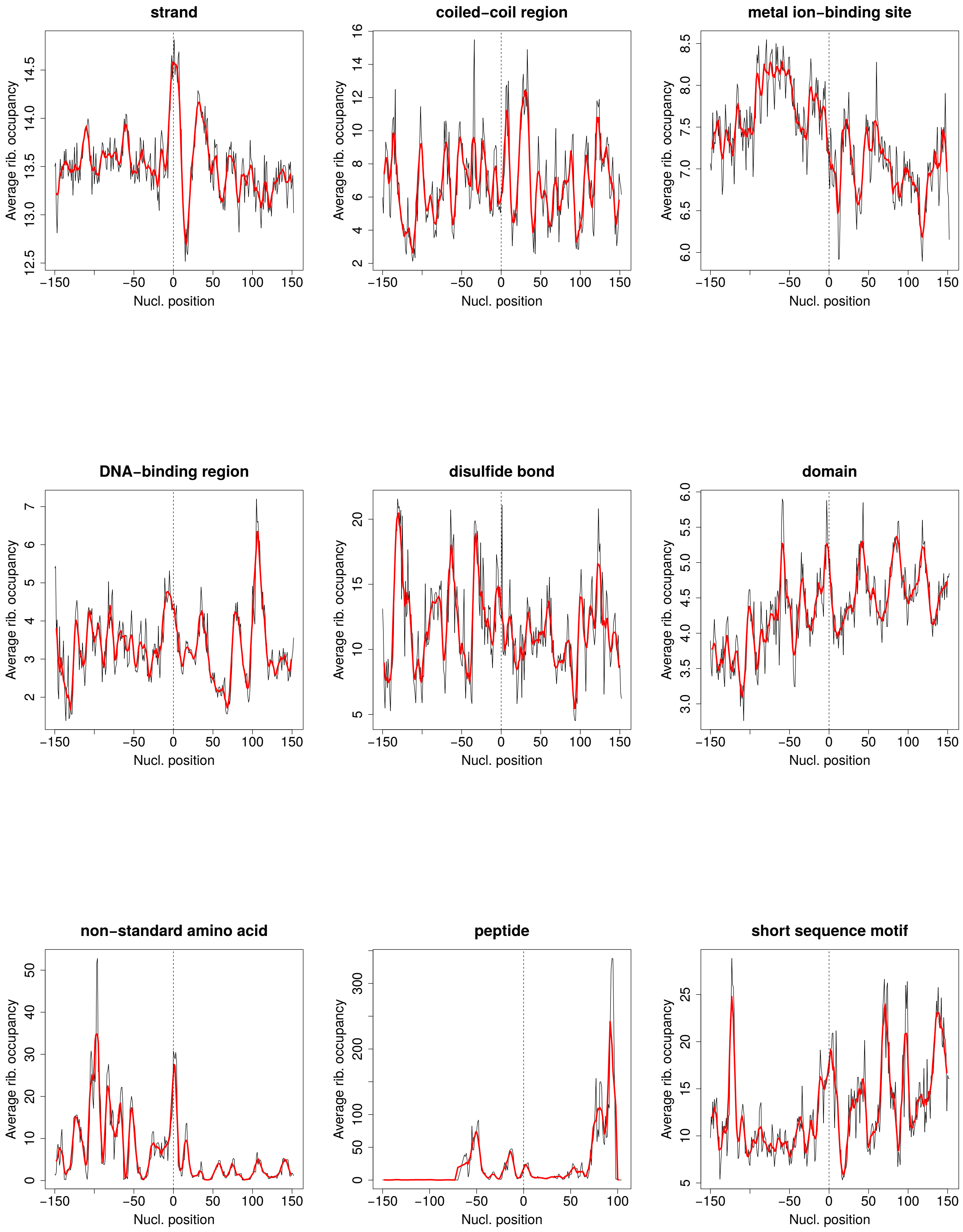
strand
coiled−coil region
metal ion−binding site
DNA−binding region
disulfide bond
domain
non−standard amino acid
peptide
short sequence motif
Average rib. occupancy
Nucl. position

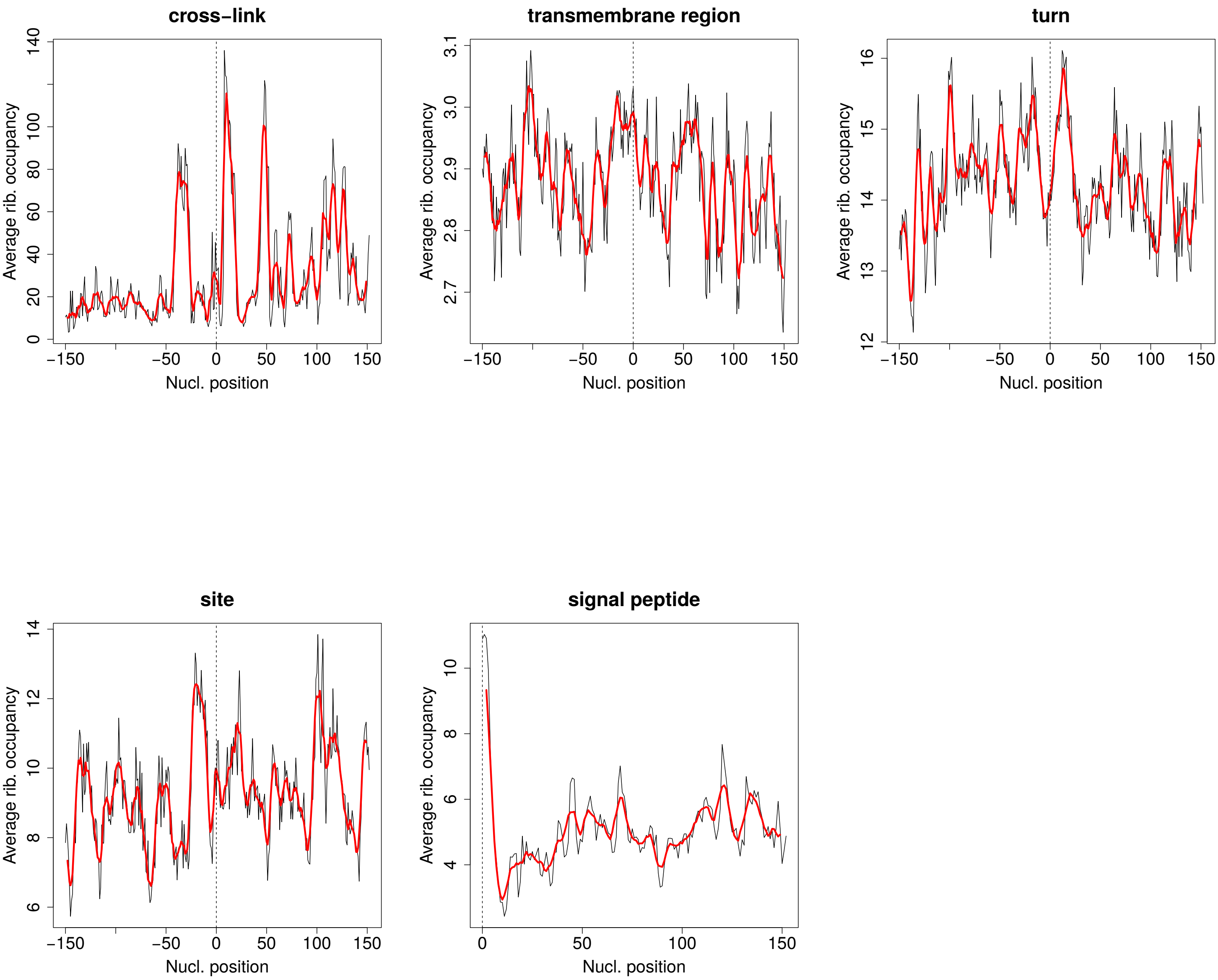
cross-link
transmembrane region
turn
site
signal peptide
Average rib. occupancy
Nucl. position

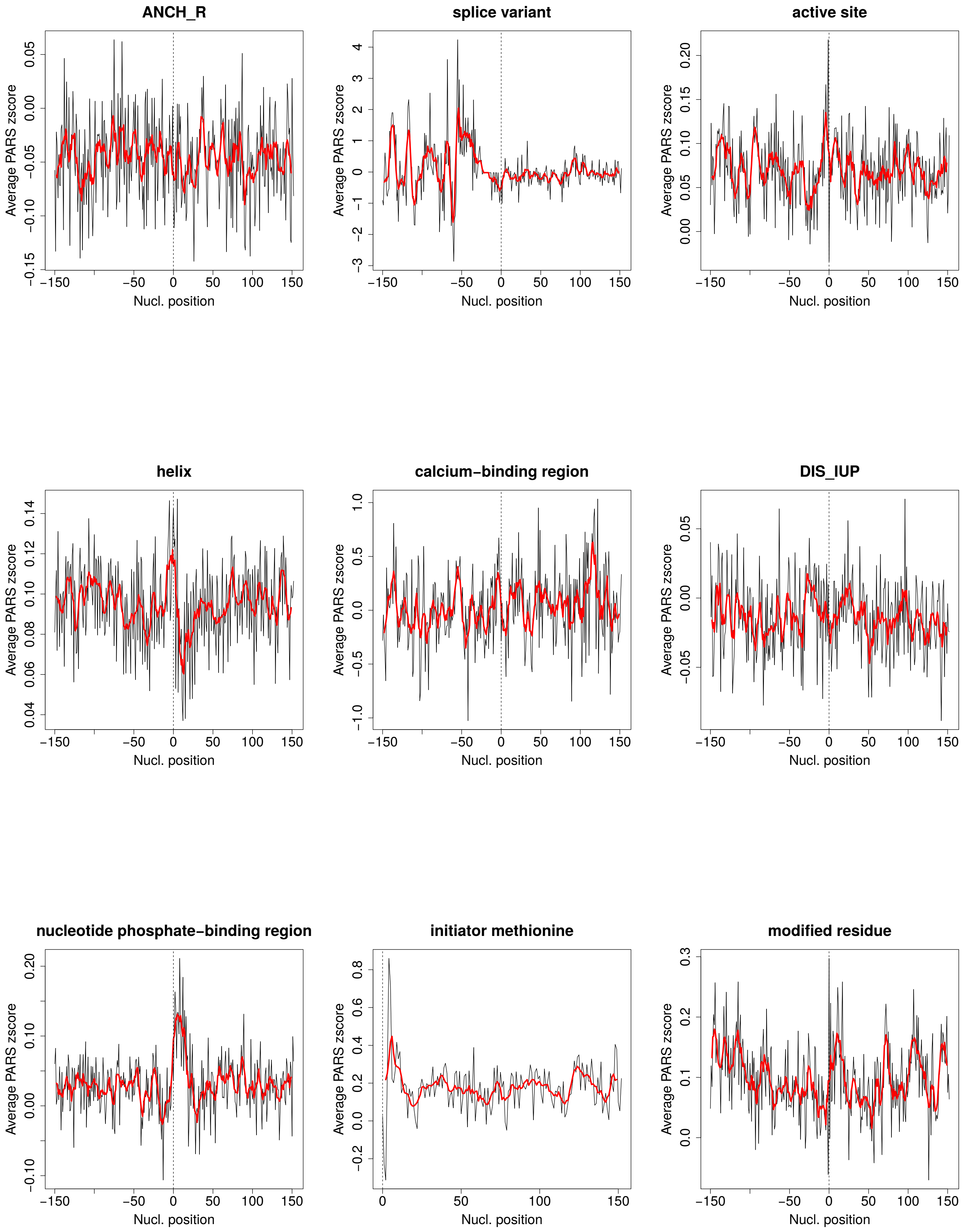

ANCH_R
Average PARS zscore
−0.15
−0.10
−0.05
0.00
0.05
−150
−50
0
50
100
150
Nucl. position
splice variant
Average PARS zscore
−3
−2
−1
0
1
2
3
4
−150
−50
0
50
100
150
Nucl. position
active site
Average PARS zscore
0.00
0.05
0.10
0.15
0.20
−150
−50
0
50
100
150
Nucl. position
helix
Average PARS zscore
0.04
0.06
0.08
0.10
0.12
0.14
−150
−50
0
50
100
150
Nucl. position
calcium−binding region
Average PARS zscore
−1.0
−0.5
0.0
0.5
1.0
−150
−50
0
50
100
150
Nucl. position
DIS_IUP
Average PARS zscore
−0.05
0.00
0.05
−150
−50
0
50
100
150
Nucl. position
nucleotide phosphate−binding region
Average PARS zscore
−0.10
0.00
0.10
0.20
−150
−50
0
50
100
150
Nucl. position
initiator methionine
Average PARS zscore
−0.2
0.0
0.2
0.4
0.6
0.8
0
50
100
150
Nucl. position
modified residue
Average PARS zscore
0.0
0.1
0.2
0.3
−150
−50
0
50
100
150
Nucl. position

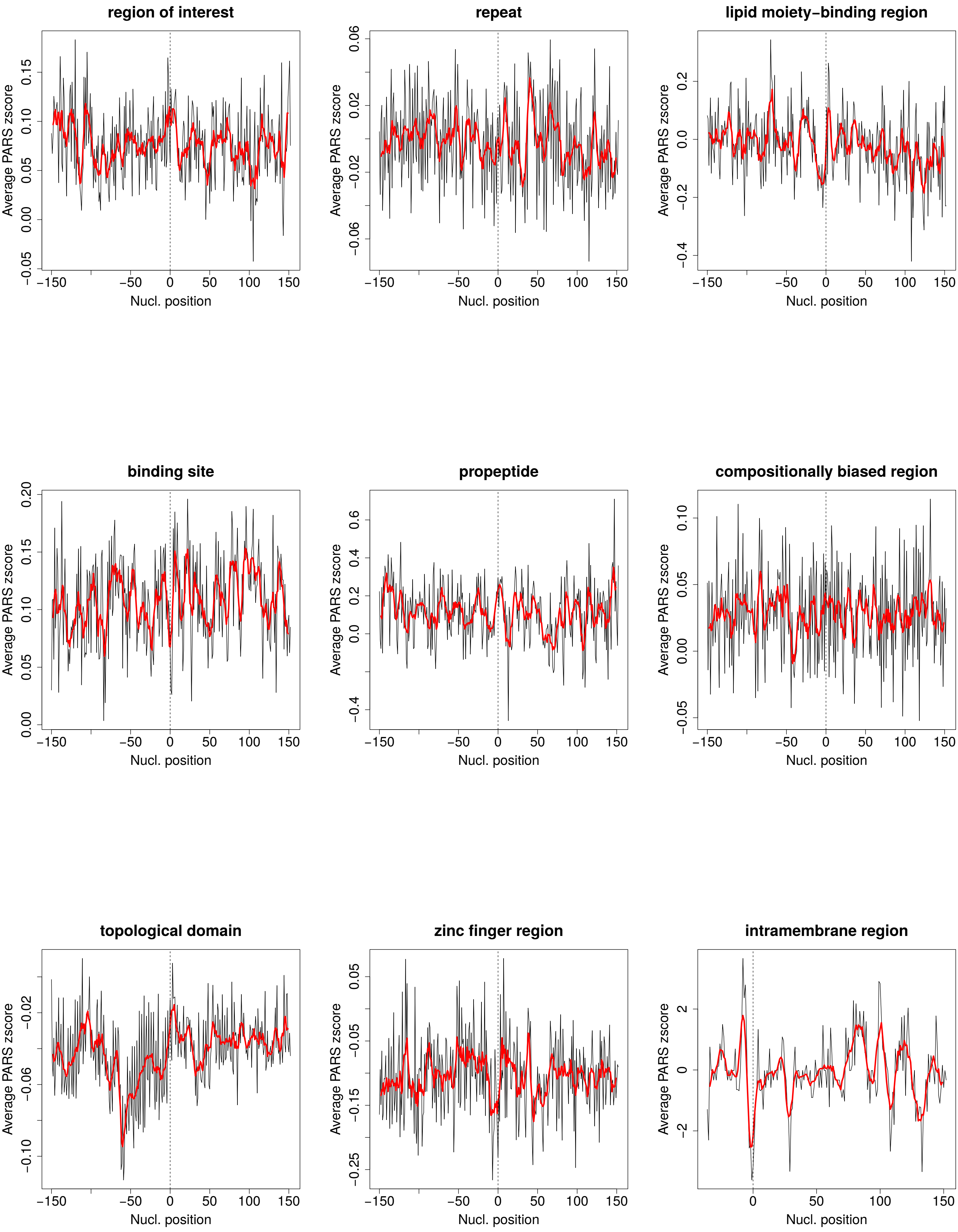

region of interest
repeat
lipid moiety−binding region
binding site
propeptide
compositionally biased region
topological domain
zinc finger region
intramembrane region
Average PARS zscore
Nucl. position
−150
−50
0
50
100
150
−0.05
0.00
0.05
0.10
0.15
0.20
−0.06
−0.02
0.02
0.06
−0.4
−0.2
0.0
0.2
0.4
0.6
−0.10
−0.25
−0.15
−2
2

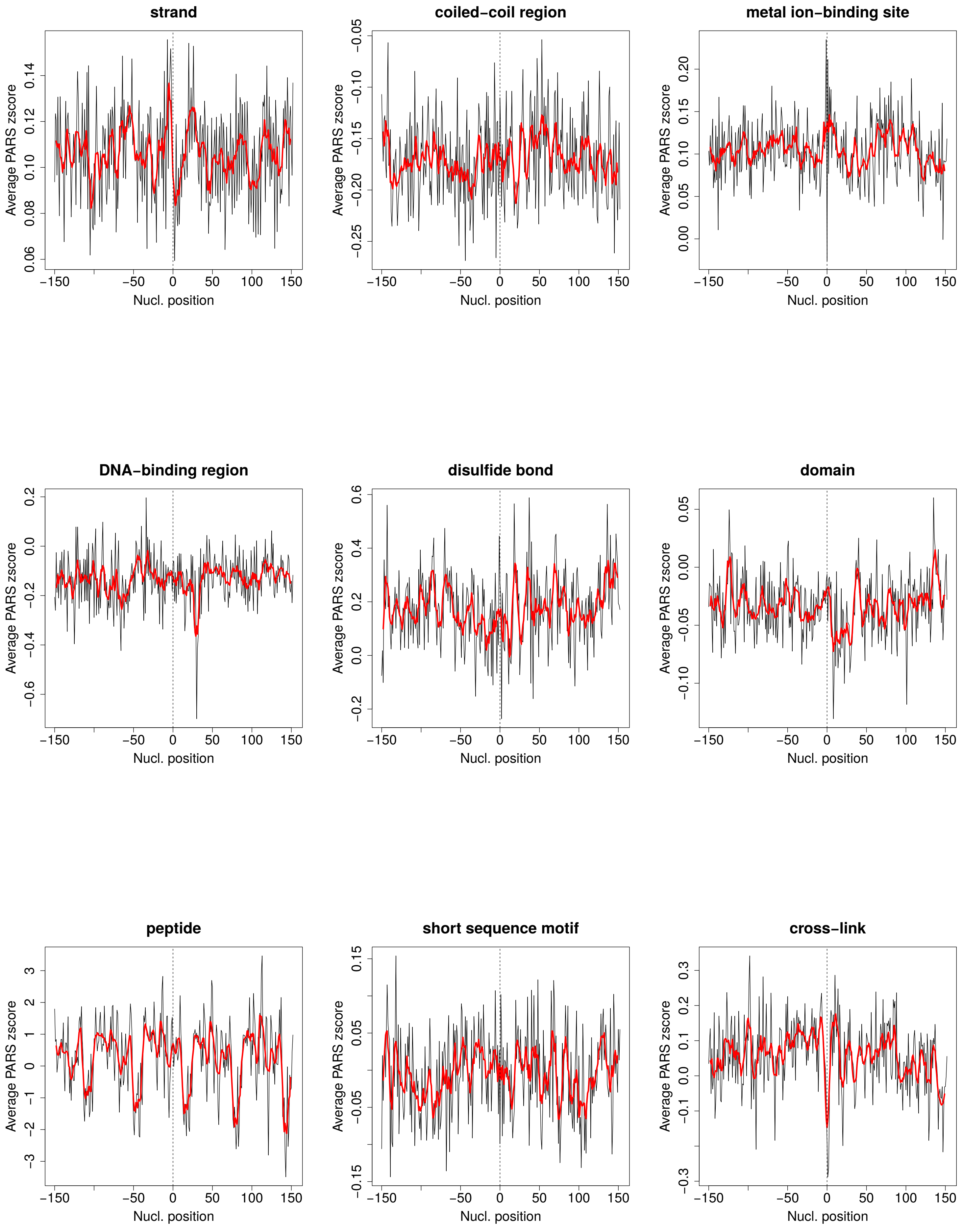
strand
coiled−coil region
metal ion−binding site
DNA−binding region
disulfide bond
domain
peptide
short sequence motif
cross−link
Average PARS zscore
Nucl. position
−150
−50
0
50
100
150

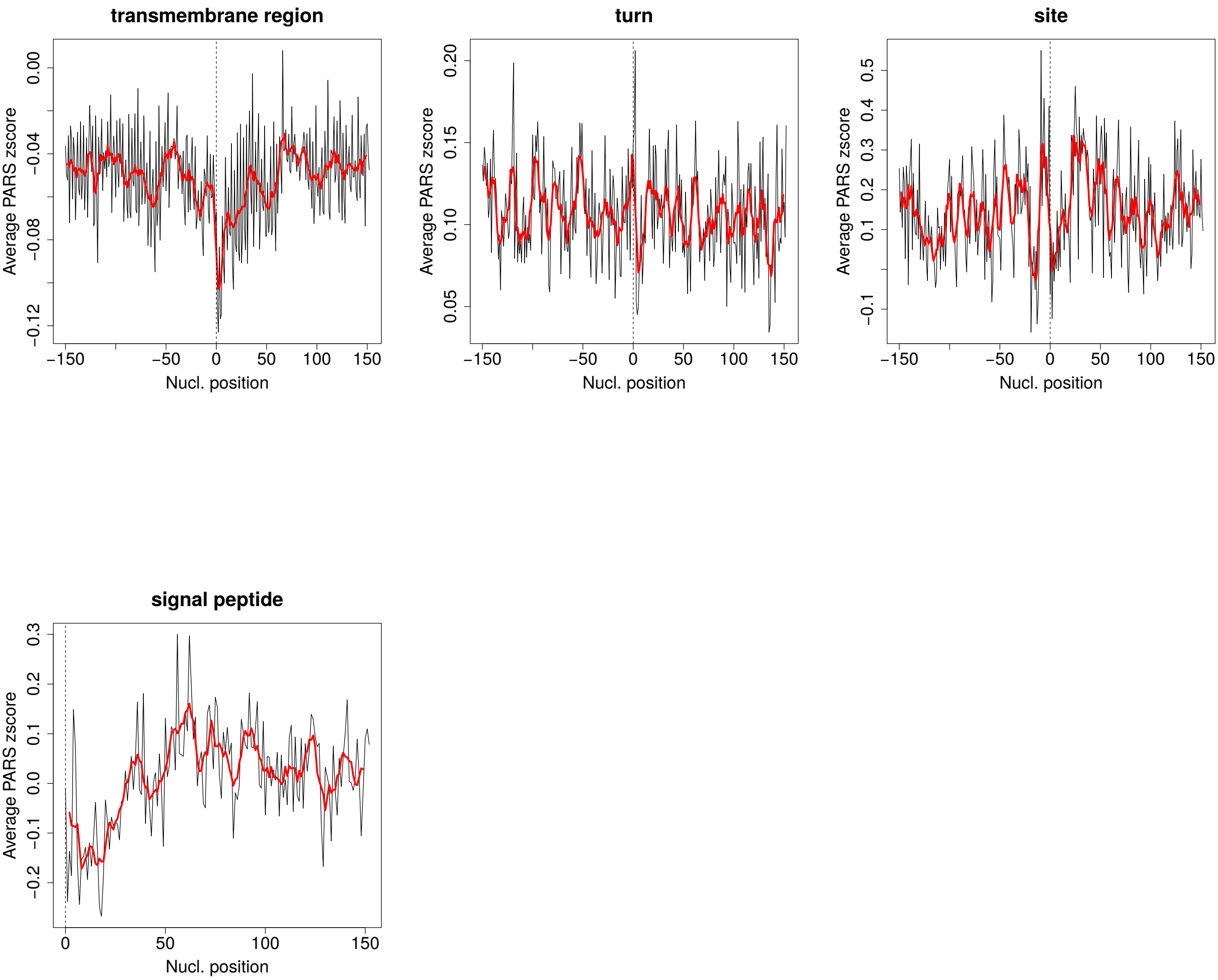

transmembrane region
Average PARS zscore
0.00
−0.04
−0.08
−0.12
−150
−50
0
50
100
150
Nucl. position
turn
Average PARS zscore
0.20
0.15
0.10
0.05
−150
−50
0
50
100
150
Nucl. position
site
Average PARS zscore
0.5
0.4
0.3
0.2
0.1
−0.1
−150
−50
0
50
100
150
Nucl. position
signal peptide
Average PARS zscore
0.3
0.2
0.1
0.0
−0.1
−0.2
0
50
100
150
Nucl. position

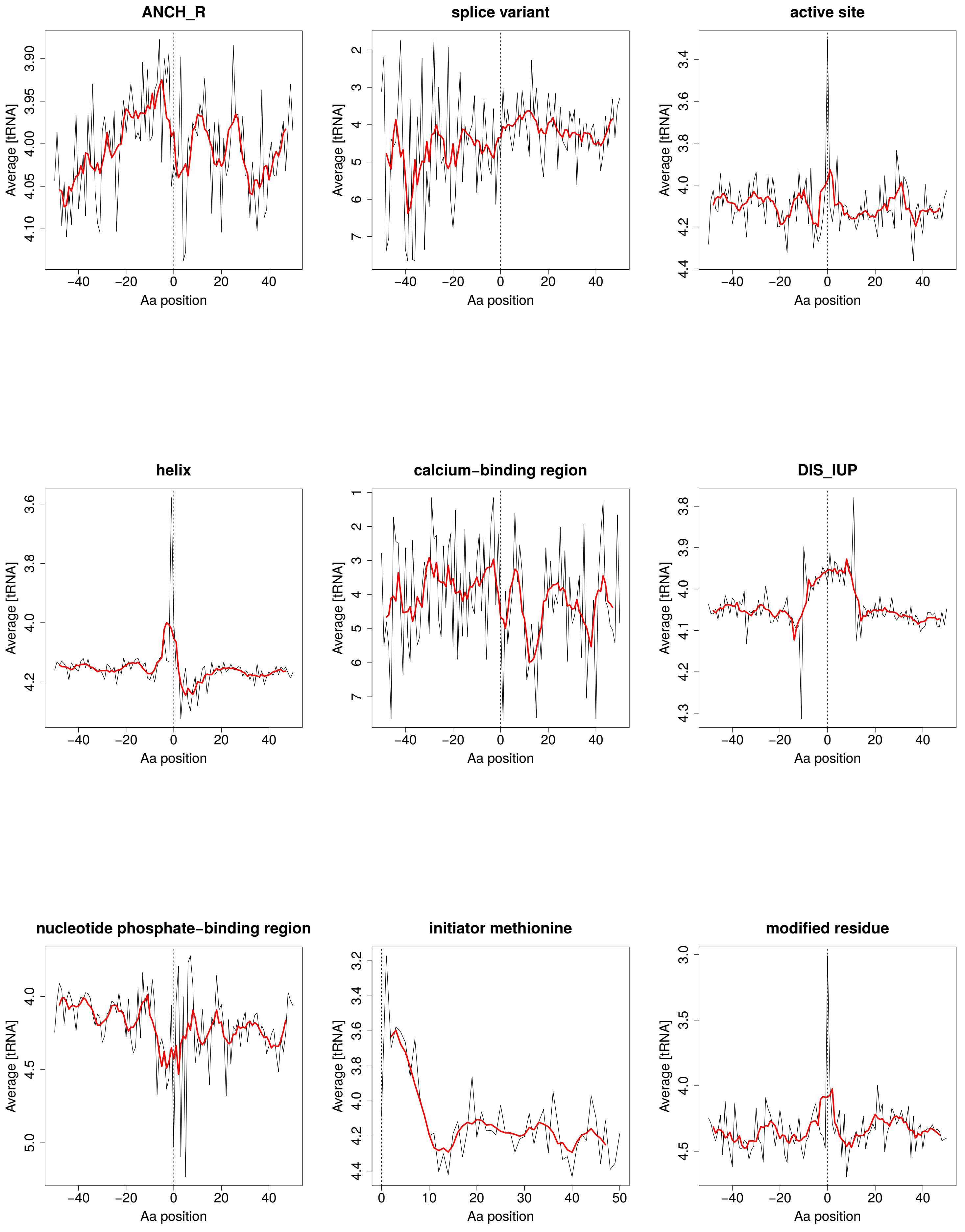

ANCH_R
splice variant
active site
helix
calcium−binding region
DIS_IUP
nucleotide phosphate−binding region
initiator methionine
modified residue
Average [tRNA]
Aa position

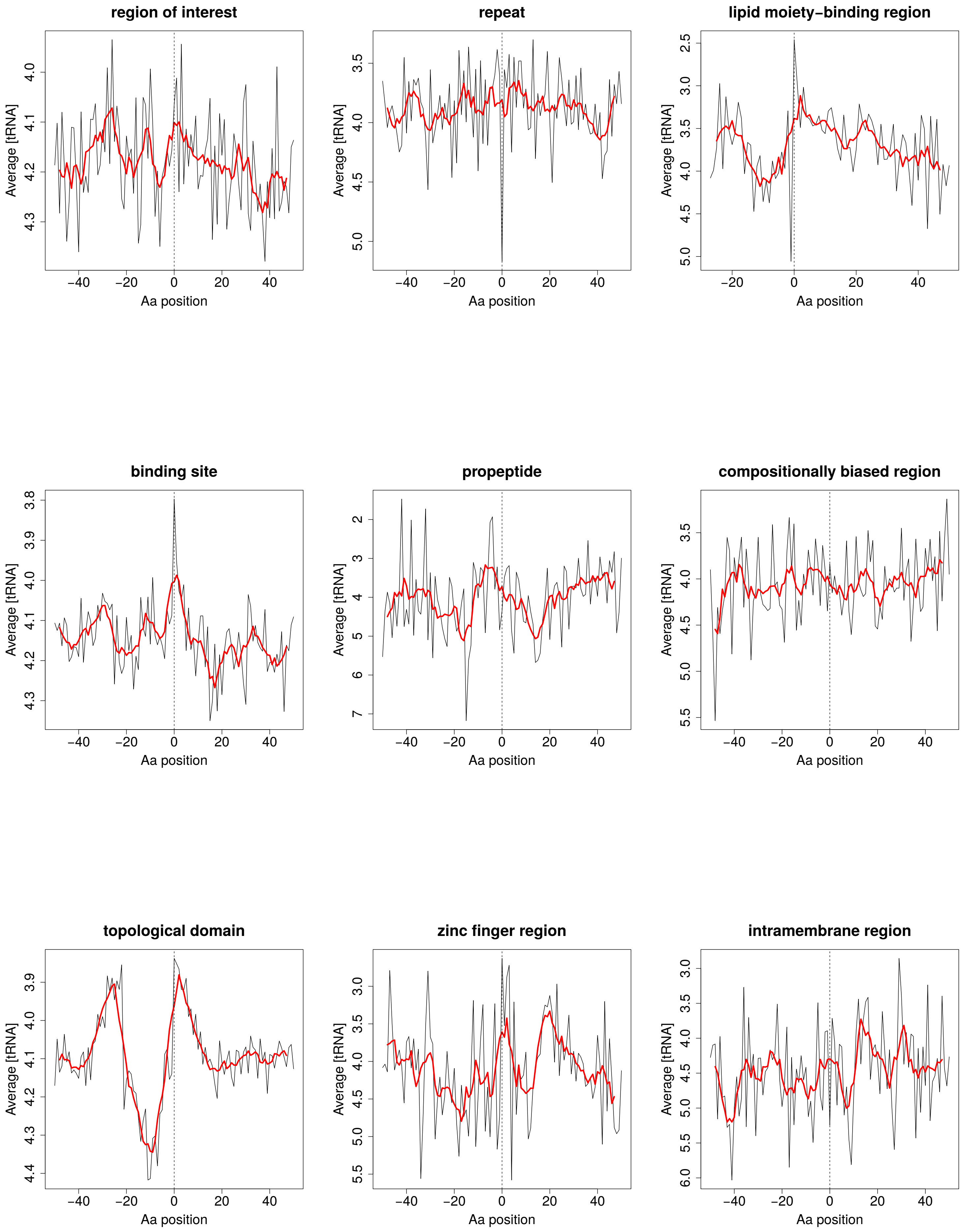

region of interest
repeat
lipid moiety−binding region
binding site
propeptide
compositionally biased region
topological domain
zinc finger region
intramembrane region
Average [tRNA]
Aa position

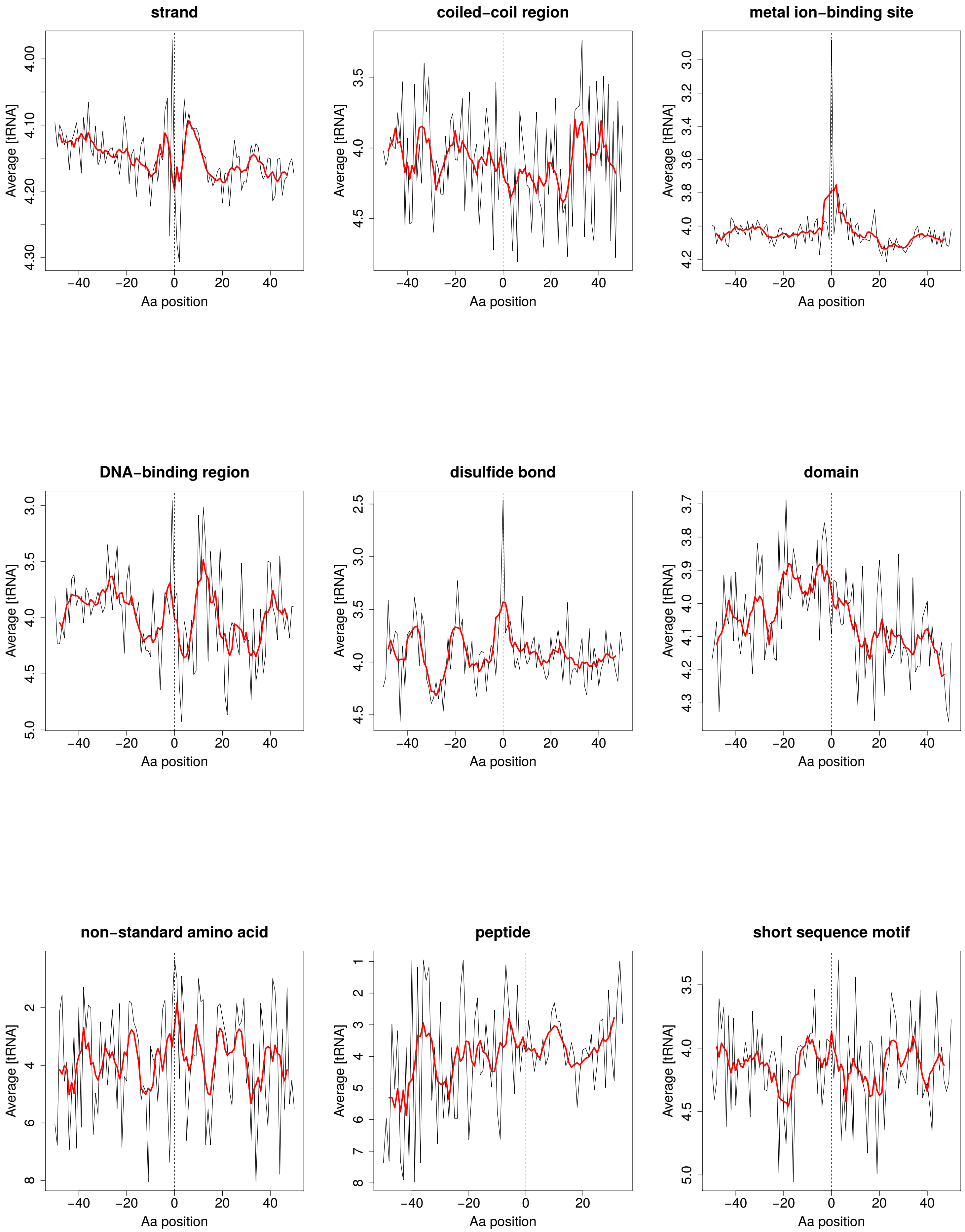
strand
coiled−coil region
metal ion−binding site
DNA−binding region
disulfide bond
domain
non−standard amino acid
peptide
short sequence motif
Average [tRNA]
Aa position

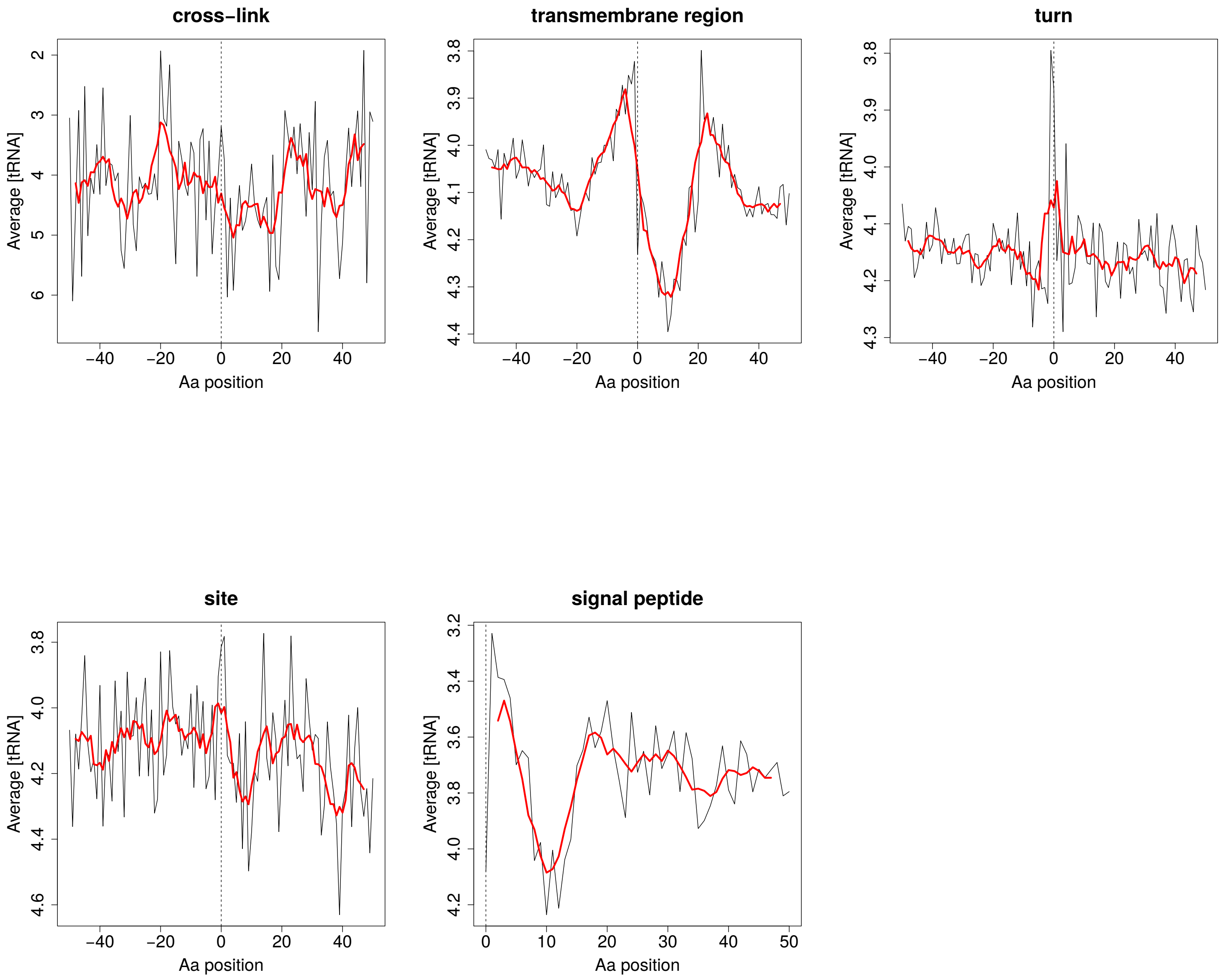
cross−link
transmembrane region
turn
site
signal peptide
Average [tRNA]
Aa position
